# Nanoparticle-Induced Breast Cancer Cell Death: The Associated Mechanisms of Seven Major Cell Death Pathways in Preclinical Models and a Cross-Validation Model

**DOI:** 10.3390/cells15070589

**Published:** 2026-03-26

**Authors:** Shirui Wang, Cuicui Chang, Rui Xu, Lizhou Wang, Bocheng Gao, Yuyang Yan, Yanju Gong, Yulin Li

**Affiliations:** 1 Main Campus, Chengdu University of Traditional Chinese Medicine, Chengdu 611137, China; 2 School of Clinical Medicine, Chengdu University of Traditional Chinese Medicine, Chengdu 611137, China; 3 Department of Clinical Medicine, Guizhou Medical University, Guiyang 550025, China; 4 School of Medical and Life Sciences, Chengdu University of Traditional Chinese Medicine, Chengdu 611137, China; 5 School of Basic Medical Sciences, Chengdu University of Traditional Chinese Medicine, Chengdu 611137, China

**Keywords:** breast cancer, nanoparticle, regulated cell death, ferroptosis, cuproptosis, disulfidptosis

## Abstract

**Highlights:**

**What are the main findings?**

- Nanoparticles induce breast cancer cell death through seven distinct regulated pathways including pyroptosis, ferroptosis, cuproptosis, and disulfidptosis via specific molecular mechanisms.
- Nanoparticle composition and surface functionalization determine the activation of specific death modalities, with mechanisms demonstrating universality across various solid tumor types.

**What is the implication of the main finding?**

- Nanoparticle-mediated non-apoptotic cell death offers critical bypass strategies to overcome apoptotic resistance that limits current clinical breast cancer treatments.
- Rational nanoplatform design based on molecular subtypes and tumor microenvironment characteristics enables personalized breast cancer therapy and facilitates clinical translation.

**Abstract:**

Breast cancer is among the most common forms of cancer in women worldwide and continues to be a major challenge in medical science and clinical care because of the complexity of its biological nature and common resistance to treatment. Nanoparticles for treating breast cancer are becoming a new-generation approach to induce the death of breast cancer cells because of their favorable physicochemical properties and excellent targeting ability. Recent studies have shown that nanoparticles can significantly increase anticancer activity by activating several cell death mechanisms, such as pyroptosis, apoptosis, necroptosis, autophagy, ferroptosis, cuproptosis, and disulfidptosis. The present review focuses on the molecular processes that lead to cell death in breast cancer models due to nanoparticle exposure. Such mechanisms have also been documented in other solid tumors, suggesting the possible universality of the cell death induced by nanoparticles. In the current review, we systematically summarize the molecular mechanisms underlying the various forms of cell death caused by nanoparticles in breast cancer cells to provide a theoretical background for the translational use of nanotechnology in precise breast cancer treatment and a cross-validation model for future mechanistic research on various types of cancer.

## 1. Introduction

Breast cancer remains among the most prevalent malignancies affecting women globally [1]. The pathogenesis of this disease is highly complex and involves interactions among genetic, lifestyle, and environmental factors [2]. Although multimodal therapies such as surgery, chemotherapy, radiotherapy, and endocrine therapy have significantly improved patient survival rates, major challenges persist, particularly drug resistance and severe treatment-related adverse effects [3]. These limitations not only compromise the efficacy of traditional treatments but also diminish patient quality of life, underscoring the urgent need to develop innovative, safer, and more efficient therapeutic strategies [4]. Breast cancer is characterized by distinct molecular subtypes, such as luminal, HER2+, and triple-negative breast cancer (TNBC), which exhibit marked differences in metabolic flux and immune landscapes [5]. For instance, a subset of TNBC cells display glutamine and cystine addiction with high expression of SLC7A11/xCT, making them vulnerable to redox-disrupting therapies such as xCT inhibition (e.g., sulfasalazine) or GLUT inhibition that induces disulfidptosis in SLC7A11-high cells [6,7,8]. Conversely, the HER2+ and luminal subtypes rely on different survival signaling pathways, which influence their threshold for undergoing apoptosis or autophagy [5]. Understanding these subtype-specific vulnerabilities is crucial for the translational design of nanoparticle-based interventions [9].

Rapid advancements in nanotechnology have ushered in a new era in breast cancer management. Owing to their unique physicochemical properties, such as tunable particle size, versatile surface functionalization, and enhanced targeting capabilities, nanoparticles have demonstrated a broad utility in drug delivery and cancer therapy [10,11]. Various nanocarriers, including polymeric nanoparticles, mesoporous silica nanoparticles, gold nanoparticles, and solid lipid nanoparticles (SLNs), have been extensively investigated as anticancer drug delivery systems. At present, nanocarriers can be roughly classified into two types: organic nanoparticles (such as polymer nanoparticles, solid lipid nanoparticles and liposomes) and inorganic nanoparticles (such as mesoporous silica, gold and metal oxide nanoparticles), which are widely studied and applied in anticancer drug delivery systems. Organic platforms are favored for their excellent biocompatibility and programmable drug release characteristics, but in recent years, inorganic nanoparticles have received increasing attention because of their unique physicochemical properties (such as photothermal conversion and inherent catalytic activity) [12,13]. These platforms have the potential to increase drug bioavailability, minimize systemic toxicity, and mitigate multidrug resistance through controlled release, increased accumulation in tumors, and improved drug stability and intracellular retention [14,15]. For instance, SLNs can encapsulate both hydrophobic and hydrophilic agents to enable controlled release and targeted delivery, thereby improving tolerability and reducing adverse effects in breast cancer treatment [16]. Furthermore, polymeric nanoparticles can be surface modified to achieve tumor-specific targeting, which further increases the therapeutic index of anticancer agents [17,18].

Inducing various forms of cell death, such as apoptosis, autophagy, and ferroptosis, is a critical strategy in the treatment of breast cancer [19]. Nanoparticles can selectively activate these cell death pathways. They can be used to directly induce tumor cell death by delivering chemotherapeutic drugs or photothermal agents, which sensitize tumors to treatment by interfering with intracellular signaling pathways, potentially delaying or reversing the onset of drug resistance [20,21]. Moreover, nanoparticles can facilitate the activation of immune cells and potentiate antitumor immune responses, representing a promising vehicle for combination immunotherapy [22].

Importantly, a critical methodological distinction must be noted when evaluating the current nanomedicine literature. While many mechanistic claims regarding nanoparticle-induced cell death remain primarily descriptive and rely on phenotypic observations or biomarker changes (e.g., ROS accumulation or altered protein expression), definitive causation requires rigorous pathway validation. Therefore, throughout this review, we critically examine representative studies to distinguish those using gold-standard pharmacological inhibitors or genetic approaches (e.g., CRISPR-Cas9 or siRNA) for confirming specific regulated cell death modalities from those drawing conclusions predominantly on the basis of biomarker profiling.

**Figure 1:**
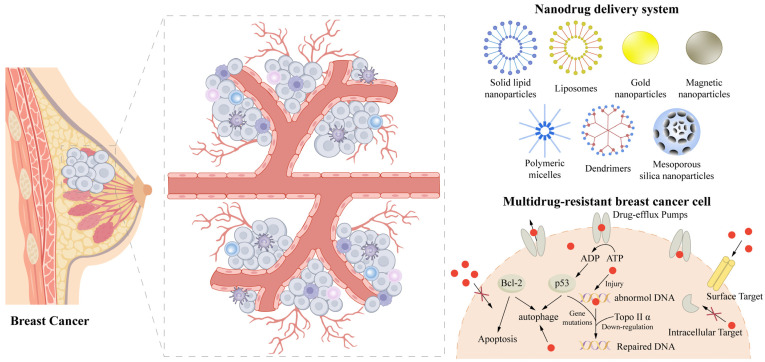
Nanoparticle-based drug delivery system for breast cancer cells. There are various types of nanocarriers, including solid lipid nanoparticles, liposomes, gold nanoparticles, magnetic nanoparticles, polymeric micelles, dendrimers, and mesoporous silica nanoparticles, which are designed to improve drug solubility, stability, bioavailability, and targeted delivery efficiency. The key drug resistance mechanisms and potential therapeutic targets of multidrug resistant breast cancer cells include drug efflux pumps, anti-apoptotic protein Bcl-2, *p53* mutations, dynamic changes in tubulin, autophagy activation, gene mutations and DNA abnormalities, down-regulation of topoisomerase IIα, surface and intracellular targets, and DNA repair enhancement. Nanotech-based delivery strategies can overcome multidrug resistance and induce tumor cell death in breast cancer by achieving targeted drug delivery, increasing intracellular drug accumulation and acting on multiple pathways. Grey spheres represent breast cancer cells, while other colored spheres represent various infiltrating immune cells (e.g., lymphocytes and macrophages). Red solid circles represent therapeutic drugs or nano-formulations. Black arrows indicate biological processes. Red cross signs (×) denote the inhibition of specific pathways or the failure of drug-target interactions.

Owing to their distinct structural and functional properties, nanoparticles offer a potential solution to the challenges of drug resistance and treatment-induced toxicity associated with traditional breast cancer therapies. However, it is crucial to note that specific TME metabolic features, which include localized hypoxia, extracellular acidity, and aberrant redox homeostasis driven by elevated glutathione (GSH), represent a biological paradox. Whereas they act as primary biological barriers limiting nanoparticle delivery, they can also be harnessed as responsive triggers to modulate local ROS balance, influence immune activation, and ultimately define the sensitivity of breast cancer cells to regulated cell death [23]. Capitalizing on this duality, the integration of nanomedicine with tumor biology is paving the way for more precise and personalized breast cancer treatments through the regulation of diverse cell death processes [24]. This review systematically summarizes the molecular mechanisms through which nanoparticles trigger different modes of cell death in breast cancer cells and discusses their applicability across various tumor subtypes. It aims to provide a theoretical framework to guide future research on nanomedicine in breast cancer treatment and facilitate the clinical translation of these novel therapeutic approaches (Figure 1).

## 2. Pyroptosis

### 2.1. Biological Characteristics of Pyroptosis and Its Role in Breast Cancer

Pyroptosis is a type of Regulated Cell Death involving the gasdermin protein family that is evolutionarily conserved from prokaryotes to mammals [25]. The term pyroptosis is derived from the Greek words “pyro” (fire) and “ptosis” (falling), implying that this form of cell death is inherently inflammatory [26]. Its initiation typically requires the cleavage of gasdermin proteins by specific proteases to release the N-terminal pore-forming domain. This domain oligomerizes within the cell membrane to form pores, disrupting membrane integrity. This disruption leads to the dissipation of ion gradients and a massive influx of water driven by osmotic pressure, ultimately resulting in cell swelling and lysis [25]. Morphologically, pyroptosis is characterized by rapid cell swelling, membrane rupture, and the release of cytosolic contents. Concurrently, mature proinflammatory cytokines, specifically IL-1β and IL-18, are secreted into the extracellular space via gasdermin pores, thereby amplifying local and potentially systemic inflammatory responses. While noncanonical pathways exist, pyroptosis is canonically mediated by inflammasomes that are activated upon the sensing of endogenous or exogenous stimuli, such as pathogen-associated molecular patterns (PAMPs) and damage-associated molecular patterns (DAMPs) [27]. Key morphological hallmarks include cellular swelling (ballooning), increased membrane permeability, and lactate dehydrogenase (LDH) release [28]. This cell death mechanism not only eliminates infected or damaged cells but also recruits immune cells via the release of inflammatory mediators. Although it serves as a crucial component of innate immunity against infection, aberrant pyroptosis is implicated in sepsis, autoimmune diseases, and neuroinflammation. Conversely, its capacity to potentiate antitumor immunity highlights its potential as a promising therapeutic target [29].

In breast cancer, pyroptosis is increasingly recognized to exert dual, context-dependent effects. It functions as a tumor suppressor by directly inhibiting tumor growth. For instance, tumor necrosis factor-α (TNF-α) induces mitochondrial dysfunction and increases mitochondrial reactive oxygen species (ROS), thereby activating the NLRP3–caspase-1–gasdermin D (GSDMD) pathway to trigger pyroptosis and suppress proliferation in MCF-7 breast cancer cells (a representative luminal A subtype model) [30]. Conversely, inflammatory mediators released during pyroptosis can activate immune cells within the tumor microenvironment (TME), contributing to the regulation of antitumor immunity. Emerging evidence suggests that NLRP3 inflammasome-mediated pyroptosis influences tumor progression and metastasis by modulating the immunosuppressive microenvironment in breast cancer [31]. This strategy is particularly critical for aggressive subtypes such as triple-negative breast cancer (TNBC), which typically exhibit a highly immunosuppressive tumor microenvironment (TME) characterized by insufficient tumor-infiltrating immune cells and immunosuppressive cytokines. Intentional induction of pyroptosis via engineered nanoparticles results in the release of abundant damage-associated molecular patterns (DAMPs), which effectively disrupt local immune tolerance and actively stimulate the maturation of dendritic cells alongside the infiltration of cytotoxic T lymphocytes. This conversion from an immunologically ‘cold’ to a ‘hot’ TME achieves robust immune activation, thereby priming the tumor for synergistic immunotherapeutic approaches [32].

At the transcriptome level, numerous bioinformatics analyses have revealed the dysregulated expression of pyroptosis-related genes in breast cancer, which is significantly associated with patient prognosis. These genes have been used to construct prognostic models that robustly predict clinical outcomes and immune status in patients with breast cancer [33,34]. These findings suggest that pyroptosis-related genes not only contribute to tumor cell death but also influence breast cancer progression by reshaping the immune microenvironment. Specifically, genes such as *KLRB1*, *CHI3L1*, *PAK7*, and *FREM1* have been implicated in breast cancer pathogenesis and therapeutic responses via the modulation of the immune microenvironment.

### 2.2. Molecular Mechanisms of Pyroptosis Activation by Nanoparticles

In breast cancer cells, nanoparticles trigger canonical and noncanonical pyroptosis through an interplay of oxidative events. Direct mitochondrial impairment and concurrent glutathione depletion force the accumulation of reactive oxygen species. This unchecked oxidative stress compromises the membrane, leading to pore formation and cellular demise [35].

**Figure 2:**
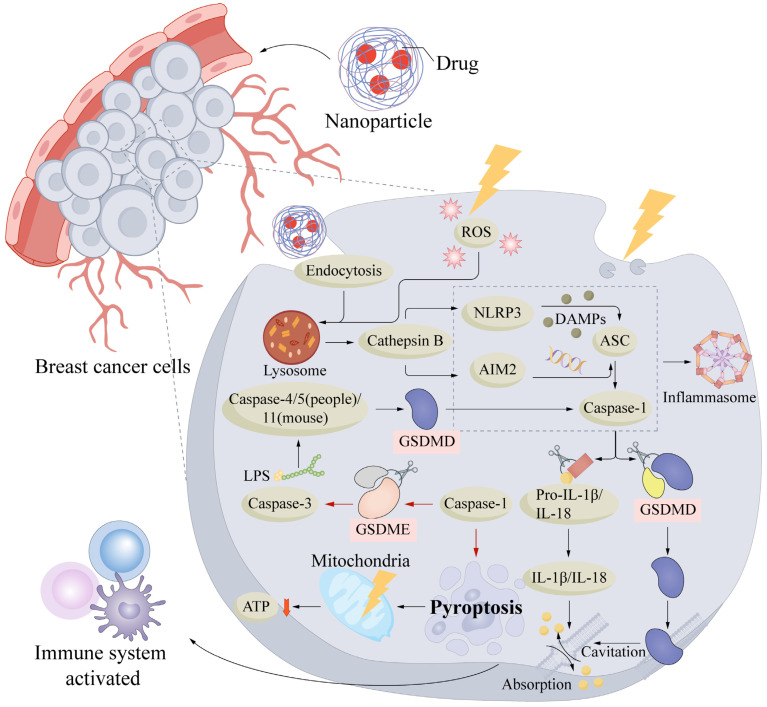
The molecular mechanism of nanoparticle-induced pyroptosis of breast cancer cells. Nanoparticles enter breast cancer cells through endocytosis or membrane damage, triggering ROS bursts, lysosomal damage or directly providing signals such as lipopolysaccharide (LPS), which then activate the classical LRR- and pyrin domain-containing protein 3 (NLRP3)-Caspase-1 pathway or the non-classical Caspase-4/5/11 pathway. These activated Caspases cleave the key executor of pyroptosis, gasdermin D (GSDMD), and its N-terminal fragment forms pores on the cell membrane, leading to the release of cell contents and inflammatory factors interleukin-1 Beta (IL-1β) and interleukin-18 (IL-18), thereby activating the immune response and forming an antitumor positive feedback loop. The downward-pointing orange arrow indicates the reduction in ATP production. Thin red arrows highlight the key underlying mechanisms and critical regulatory steps. Black arrows indicate biological processes.

The molecular mechanisms underlying nanoparticle-induced pyroptosis in tumor cells are generally categorized into two primary pathways. The canonical pathway involves the activation of the NLRP3 inflammasome, which subsequently triggers caspase-1 activation and the cleavage of gasdermin D (GSDMD) to form membrane pores. In contrast, the noncanonical pathway is typically initiated by DNA damage or mitochondrial dysfunction, leading to caspase-3 activation and the subsequent cleavage of gasdermin E (GSDME). Nanoparticles can modulate these pathways through distinct mechanisms: they may directly activate signaling cascades via their intrinsic physicochemical properties, function as delivery vehicles for pyroptosis inducers, or promote the accumulation of reactive oxygen species (ROS) as precursors to the pyroptotic program. Furthermore, pyroptosis facilitates the release of damage-associated molecular patterns (DAMPs), thereby increasing tumor immunogenicity and eliciting potent antitumor immune responses [35] (Figure 2).

Pyroptosis can be triggered by various nanoplatforms, including metal-based nanoparticles and drug-loaded systems. With respect to the canonical pathway, metal-based nanoplatforms such as ACS-Z-P and BSM have been shown to increase intracellular ROS levels and activate the NLRP3 inflammasome. This activation leads to caspase-1-mediated GSDMD cleavage, resulting in membrane pore formation and the release of IL-1β upon near-infrared (NIR) or ultrasound stimulation, respectively. Notably, these therapeutic strategies have demonstrated the ability to increase CD8+ T-cell infiltration and suppress distant metastasis in a 4T1 breast cancer model (a widely used triple-negative breast cancer model) [36,37]. CS-HAPATO, a drug-loaded nanocarrier, induces pyroptosis by encapsulating atorvastatin (ATO) within CS-modified HAP and targeting tumor cells via CD44. Under acidic conditions, it releases Ca^2+^ and ATO, activates the NLRP3 inflammasome–caspase-1 axis, and promotes GSDMD cleavage. Notably, while these mechanisms are highly suggestive, the activation of the NLRP3–caspase-1 axis in this model was characterized primarily by descriptive biomarker profiling, such as GSDMD cleavage and cytokine release, rather than definitive rescue using gold-standard pharmacological inhibitors [38]. In the noncanonical pathway, TiO–F spindles generate ROS under low-intensity ultrasonic cavitation and activate caspase-3 in an inflammasome-independent manner. Caspase-3 then cleaves GSDME, which leads to pore formation and pyroptosis-associated immune activation. In the 4T1 model (a widely used triple-negative breast cancer model), this strategy synergizes with immune checkpoint blockade and markedly reduces both the primary tumor burden and the rechallenge tumor burden, indicating that GSDME-mediated pyroptosis can facilitate the remodeling of the immune microenvironment in breast cancer [39]. PLGA biomimetic granules coated with a breast cancer cell membrane were coloaded with photothermal ICG and the demethylating agent DAC, which upregulated GSDME expression in tumor cells. Upon near-infrared irradiation, ICG-mediated heating promoted cytochrome c release and activated caspase-3, leading to GSDME cleavage and noncanonical pyroptosis in 4T1 cells (a widely used triple-negative breast cancer model). This strategy also induced the release of DAMPs and systemic antitumor immunity [40]. The release of DAC in neutrophil-disguised nanomedicines increases the expression of GSDME in tumor cells, whereas IR820 generates a photothermal effect upon near-infrared irradiation. This activation leads to the activation of caspase-3, which leads to the cleavage of GSDME and pore formation on the plasma membrane, which results in pyroptosis in breast cancer cells [41]. In summary, the nanoparticle composition and design can influence the ability to induce pyroptosis as well as the specific pathway used by breast cancer cells, thus determining antitumor efficacy.

Another significant group of nanoplatforms comprises carbon-based nanomaterials that have demonstrated the potential to trigger pyroptosis. For instance, graphene oxide can be internalized into cells through phagocytosis and activate NADPH oxidase, which leads to lipid peroxidation in the plasma membrane. Subsequently, phospholipase C activation, intracellular Ca^2+^ release, and ROS formation in mitochondria occur. This cascade causes NLRP3 inflammasome formation and caspase-1 activation, ultimately resulting in the pyroptosis of Kupffer cells (KCs) through the cleavage of GSDMD [42]. Carbon-based nanoparticles may be cytotoxic because they induce lysosome dysfunction and trigger pyroptosis-related inflammasome pathways [43]. Thus, the surface chemistry and structure-related properties of carbon-based nanomaterials are critical determinants of pyroptosis induction. Overall, the effects of various nanoparticles on pyroptosis activation depend on the particle size, morphology, surface chemistry, and intracellular location. For example, biomimetic NPs may induce intracellular Ca^2+^ buildup facilitated by cell membrane coating, which causes mitochondrial injury, caspase-3 activation, and, subsequently, GSDME-mediated pyroptosis. Complex polymeric nanoparticles responsive to ROS can release glucose oxidase upon exposure to high-ROS conditions enabled by thioacetal-crosslinked shells, thereby inducing pyroptosis due to oxidative stress and glucose deprivation [35]. Moreover, pH-sensitive or enzyme-sensitive nanoparticles can deliver drugs or active ions to acidic tumors or lysosomes to achieve more precise activation of signaling cascades associated with pyroptosis [44,45]. For example, pH-responsive CaNMs trigger a burst release of Ca^2+^ under acidic conditions, causing mitochondrial Ca^2+^ overload and activating caspase-3, which cleaves GSDME to induce pyroptosis. MCPP, which is degraded more rapidly in a mildly acidic microenvironment, amplifies ROS upon light stimulation and similarly induces pyroptosis via the GSDME pathway. GOx-Mn/HA dienzymatic nanoparticles consume glucose to generate H_2_O_2_, whereas Mn nanozymes catalyze ROS production to activate the NLRP3–caspase-1–GSDMD pathway, ultimately leading to pyroptosis [46].

Mitochondrial damage is also a critical driver of nanoparticle-induced pyroptosis. CS-HAPATO nanoparticles release Ca^2+^ and atorvastatin in the tumor microenvironment, synergistically causing mitochondrial Ca^2+^ overload and dysfunction. Consequently, a surge of mitochondrial ROS facilitates the production and cytosolic release of oxidized mitochondrial DNA fragments, which are sensed by the NLRP3 inflammasome, leading to caspase-1 activation and GSDMD cleavage. This process culminates in pyroptosis followed by the release of inflammatory mediators [38]. Photosensitizers can be delivered to mitochondria via anhydrase-responsive nanocarriers, causing mitochondrial ROS generation and inducing pyroptosis along with its associated immune response [47]. CSEPP nanoparticles release Ca^2+^ and H_2_S to synergistically induce mitochondrial dysfunction and intracellular oxidative stress, thereby activating caspase-1, promoting GSDMD cleavage, and inducing pyroptosis in tumor cells [48]. Nanomotors achieve mitochondrial accumulation via surface-modified triphenylphosphine groups. They catalyze nitric oxide (NO) production inside mitochondria, reduce the mitochondrial membrane potential, and facilitate cytochrome c release upon ROS stimulation. This triggers caspase-3 activation, which cleaves GSDME to generate membrane pores, eventually leading to cell swelling, membrane rupture, and the release of inflammatory mediators that are characteristic of pyroptosis [49].

In addition to causing mitochondrial damage, nanoparticles may also increase oxidative stress by lowering the level of intracellular glutathione (GSH) and increasing the level of ROS. MCPP NPs are dual-responsive (ROS/GSH) nanoplatforms capable of delivering chemotherapeutic drugs and photosensitizers, thereby triggering caspase-3 activation and GSDME cleavage and initiating tumor cell pyroptosis under ROS burst conditions [50]. HPPH-ss-NPs are responsive to GSH; they deliver DEC and reduce the level of GSH upon 660 nm irradiation while simultaneously inducing ROS saturation and mitochondrial dysfunction. This leads to cascades of caspase-3 activation, GSDME cleavage, and rapid pyroptosis induction in breast cancer cells [51].

Nanoparticles can also be used to synergize with photothermal therapy and photodynamic therapy. These modalities can activate the caspase-3/GSDME pathway to induce pyroptosis, leading to immunogenic cell death, the release of inflammatory mediators and antigens, and robust antitumor immune responses that improve therapeutic efficacy in patients with breast cancer [52,53]. The combination of BRD4-PROTAC nanoparticles with photosensitizers has been used to achieve light-induced pyroptosis and potent suppression of lung metastasis in breast cancer models [54]. Collectively, these studies offer mechanistic insights and potential therapeutic targets for precision breast cancer treatment.

The activation of pyroptosis by diverse nanoparticles may also interact with other types of Regulated Cell Death initiated by the same platforms. For instance, ROS-sensitive PIC nanoreactors induce GSH depletion and inhibit glutathione peroxidase 4 (GPX4) activity. This inhibition is driven by the severe oxidative stress and reactive oxygen species (ROS) generated during GOx-catalyzed glucose oxidation. Concurrently, they stimulate caspase-3 to cleave GSDME and initiate pyroptosis while simultaneously activating ferroptosis, thus initiating two distinct types of controlled regulated cell death (RCD) [35]. Other nanoparticles induce pyroptosis and immunogenic cell death, which increases the immune response in the tumor microenvironment and can serve as a rationale for combined immunotherapy approaches [46].

Distinct mechanisms exist through which various classes of nanoparticles can activate pyroptosis, and these nanoplatforms have been used in a variety of tumor models. To induce pyroptosis more precisely, several regulatory interventions may be combined to increase therapeutic efficacy. For instance, HMCe6HPBCS-5, a nanoplatform, integrates drug loading, membrane coating, enzyme responsiveness, GSH depletion, and redox modulation. It generates ROS via the photodynamic activity of Ce6 and synergizes with CS-5 to inhibit GPX4, thereby amplifying oxidative stress, caspase-3 activation, and GSDME cleavage. This induces the formation of membrane pores and morphological alterations characteristic of pyroptosis in 4T1 breast cancer cells (a widely used triple-negative breast cancer model). The immunogenic cell death signals associated with pyroptosis promote dendritic cell maturation and CD8+ T-cell infiltration, which significantly inhibit primary tumors, distant metastases, and lung metastasis in the 4T1 model [55]. However, importantly, while the immunocompetent 4T1 model is highly valuable for evaluating these immune responses, its intrinsic immune environment may not fully recapitulate the profound and complex immunosuppressive nature of human breast cancer, suggesting that these robust therapeutic outcomes should be interpreted with cautious optimism. Accordingly, the next generation of nanoparticles must prioritize their physicochemical characteristics, targeting efficacy, and intracellular release behavior to optimize the clinical translation of pyroptosis-inducing strategies and advance the treatment of breast cancer.

## 3. Apoptosis and Necroptosis

### 3.1. Differentiation Between Apoptosis and Necrotizing Apoptosis and Its Significance in the Treatment of Breast Cancer

Apoptosis involves noninflammatory, tightly controlled mechanisms of Regulated Cell Death. The apoptotic process is mediated by signaling cascades that initiate effectors such as caspases, which promote membrane blebbing, chromatin condensation, cellular shrinkage, DNA fragmentation into nucleosomal fragments, and the formation of apoptotic bodies. It is particularly critical that membrane integrity is not significantly compromised, which facilitates the maintenance of tissue homeostasis and physiological cell turnover [56]. Inducing apoptosis in tumor cells is a fundamental therapeutic mechanism used in breast cancer therapy (e.g., chemotherapy and radiotherapy). Nevertheless, it has been suggested that cancer cells may develop resistance to drugs by blocking apoptotic signaling, thereby compromising the efficacy of therapy [57].

Unlike apoptosis, necroptosis is a type of programmed necrosis involving receptor-interacting protein kinase 1 (RIPK1), receptor-interacting protein kinase 3 (RIPK3), and mixed lineage kinase domain-like protein (MLKL). Necroptosis is characterized by cell swelling, membrane disruption, and leakage of intracellular contents. It is highly proinflammatory and may also activate the immune system, thereby eliciting inflammatory processes [58]. These proinflammatory properties contribute to the significant role of necroptosis in the context of antitumor immunity. Notably, when tumor cells escape apoptosis and acquire treatment resistance, necroptosis offers an alternative cell death pathway to eliminate cancer cells [59,60].

In the treatment of breast cancer, necroptosis has been proposed as an alternative cell death program when apoptosis fails. Apoptosis reduces tumor burden via the noninflammatory elimination of dying cells and represents one of the primary modes of cell death triggered by conventional chemotherapeutic agents, including doxorubicin [61]. However, the resistance of tumor cells to apoptosis limits the efficacy of treatments that rely solely on apoptosis induction. Necroptosis triggers the release of damage-associated molecular patterns (DAMPs) and elicits an antitumor immune response, potentially leading to enhanced immunotherapy outcomes [62].

Apoptosis has long been regarded as a noninflammatory process that involves maintaining tissue homeostasis and eliminating tumor cells. In certain instances, apoptosis may engage the immune system by releasing DAMPs and accelerating dendritic cell maturation and T-cell responses. In contrast, necroptosis is typically highly inflammatory, as membrane rupture and DAMP release can trigger inflammation and augment antitumor immunity. However, the beneficial effects of necroptosis on immune responses in tumors such as breast cancer depend on the tumor microenvironment and are context specific; indeed, necroptosis has also been implicated in immunosuppression and metastasis. Thus, the immune-activating properties of necroptosis should be harnessed and controlled with caution in a context-dependent manner in breast cancer treatment [63,64]. Both apoptosis and necroptosis have distinct mechanisms and therapeutic potentials, suggesting that future approaches should consider the coordinated regulation of both (particularly in conjunction with nanotechnology) to achieve effective, precise, and immunoactivated breast cancer therapy. For instance, photothermal therapy using nanoparticles or the combination of nanocarriers delivering chemotherapeutic agents with immunomodulators can induce apoptosis and necroptosis simultaneously, facilitating the elimination of a greater tumor burden and activation of the immune system [65].

To avoid conceptual redundancy in evaluating these therapies, clearly delineating the mechanistic boundaries among highly inflammatory cell death, such as pyroptosis, necroptosis, and ferroptosis, is imperative. While all these pathways culminate in membrane rupture and the release of damage-associated molecular patterns [27,35,62], their executioner mechanisms are distinct. Pyroptosis is uniquely driven by the pore-forming activity of the gasdermin protein family [25,28,29]. Necroptosis is executed exclusively by MLKL oligomerization and membrane translocation [35,66]. It typically functions as a failsafe alternative when apoptosis is blocked [59,60,61]. Ferroptosis involves no pore-forming proteins but is defined by metabolic collapse in which iron-catalyzed lipid peroxidation overwhelms the antioxidant axis [67,68,69].

### 3.2. Signaling Pathways for Nanoparticle-Induced Apoptosis

Nanoparticles hijack both the intrinsic mitochondrial and extrinsic death receptor pathways to strip tumor cells of their apoptotic resistance. They compromise membrane permeability and unleash reactive oxygen species; this abrupt stress stalls the cell cycle, ultimately leading to cell death [70,71].

Nanoparticle-induced apoptosis in breast cancer cells involves primarily two canonical pathways: the mitochondrial (intrinsic) pathway and the death receptor (extrinsic) pathway. In the intrinsic pathway, disruption of the mitochondrial membrane potential and alterations in the balance between pro- and antiapoptotic proteins promote the release of cytochrome c (cyt c) into the cytosol, which activates downstream caspase-9 and caspase-3 [70]. For instance, paclitaxel–triphenylphosphine (PTX–TPP) prodrugs target mitochondria, disrupt membrane potential, and increase outer membrane permeability, thereby promoting cytochrome c release and activating intrinsic caspase cascades. This approach markedly increases mitochondria-mediated apoptosis in MCF-7 breast cancer cells (a classic luminal A model) [72]. Because luminal breast cancers are typically treated with endocrine therapies but often develop resistance through the activation of compensatory survival pathways [73], the use of nanomedicines to directly trigger intrinsic apoptosis represents a critical precision strategy to overcome such resistance in this specific subtype [74]. ZnO nanofluids induce apoptosis in breast cancer stem-like cells primarily by inhibiting the JAK/STAT signaling pathway and downregulating the expression of antiapoptotic proteins such as Mcl-1 and Bcl-XL [75]. Infrared laser–excited conjugated polymer nanoparticles modulate TRPA1 channels to inhibit Ca^2+^–calmodulin complex formation, suppress the antiapoptotic protein Mcl-1, and promote ROS-mediated apoptosis, thereby accelerating cancer cell death [71]. In contrast, the extrinsic pathway is initiated by ligand–receptor engagement (e.g., via TRAIL signaling), which activates caspase-8 and subsequently caspase-3 to execute apoptosis [74,76]. Nanoparticle carriers upregulate TRAIL expression and act synergistically to induce caspase-8 cleavage, thereby bypassing resistance to TRAIL-induced apoptosis and increasing therapeutic efficacy [74]. Furthermore, mPEG-PCL-DDAB nanoparticles loaded with siRNA can be surface modified to simultaneously silence insulin-like growth factor-1 receptor and integrin αvβ3, leading to cell cycle arrest and apoptosis in breast cancer cells and showing multitarget synergy [77,78]. In general, nanoparticles may activate the intrinsic apoptotic cascade through the mitochondrial pathway, along with promoting extrinsic apoptosis by enhancing death receptor signaling, highlighting their multifunctional proapoptotic effects in breast cancer cells.

In breast cancer, nanoparticles can facilitate ROS production, perturb membrane permeability, and induce cell cycle arrest. Treatment with SLNPs can significantly increase intracellular ROS. As a signaling molecule, ROS can trigger the JNK and p38 MAPK pathways, initiating apoptosis [79]. ZnO nanoparticles may stimulate intrinsic apoptosis signaling via oxidative stress, followed by alterations in cell cycle regulatory protein expression that lead to cell cycle arrest [80]. Silver nanoparticles (AgNPs) disrupt membrane integrity by generating ROS and lipid peroxidation, leading to increased membrane permeability and the occurrence of apoptosis and necrosis [81]. Other nanoparticles may damage membrane structure directly and synergize with ROS to induce apoptosis via mechanical injury. Specifically, tBTOma-NPs can spontaneously assemble within the acidic tumor microenvironment. Once excited via ultrasound, the assembled structures produce increased levels of ROS compared with their monodisperse analogs and form submicron structures surrounding 4T1 cells (a widely used triple-negative breast cancer model), thus mechanically damaging them and inducing apoptosis [82]. Another mechanism of growth inhibition is cell cycle arrest; for instance, composite nanoparticles containing paclitaxel can arrest cell division in breast cancer cells [58]. Similarly, mPEG-PCL-DDAB nanoparticles loaded with siRNA targeting *IGF-1R* can induce cell cycle arrest in MCF-7 cells (a representative luminal A subtype model) [77]. Overall, nanoparticles can suppress breast cancer cell proliferation and promote cell death by inducing ROS accumulation, driving oxidative stress and membrane dysfunction, and promoting cell cycle arrest, which together amplify pro-apoptotic signaling.

Emerging evidence suggests that when combined with immunostimulatory therapy, chemotherapy exerts a synergistic effect on tumor cell apoptosis. Specifically, IONP-DOX-PolyIC-EBP nanoparticles target endoglin—which is overexpressed in triple-negative breast cancer—via an endoglin-binding peptide. In the acidic tumor microenvironment, both doxorubicin (DOX) and polyinosinic acid (PolyIC) are liberated. While DOX induces DNA damage and immunogenic cell death (ICD), PolyIC activates the TLR3 pathway, leading to increased dendritic cell maturation and CD8+ T-cell activation. These components act synergistically to promote apoptosis in tumor cells, a conclusion supported by descriptive molecular changes, although further causal validation with specific inhibitors remains a subject for future investigation [83]. Similarly, the combination of chemotherapy and photodynamic therapy synergistically induces apoptosis. Biomimetic nanoplatforms facilitate dual targeting of the tumor vasculature and malignant cells through cell membrane camouflage, concurrently delivering photosensitizers and chemotherapeutics. Upon laser irradiation, these platforms generate reactive oxygen species (ROS) and accelerate drug release, thereby inducing apoptosis, damaging the vascular endothelium, and preventing the relapse of triple-negative breast cancer [84]. A recent study reported a multifunctional nanomedicine system comprising oxidative treatments and immune stimulation, which exhibited superior efficacy in increasing apoptosis and inducing ICD. IR783 nanoparticles are internalized by tumor cells, where NQO1 catalyzes β-lapachone to produce ROS. Concurrently, CUDC-101 inhibits HDAC and EGFR, further exacerbating DNA damage. ROS-driven breakdown of IR783 facilitates drug release and elicits hallmarks of ICD, including calreticulin exposure, HMGB1 release, and ATP depletion [85].

Collectively, nanoparticles induce apoptosis in breast cancer cells through multiple mechanisms, including single signaling pathway modulation and synergistic strategies that integrate chemotherapy, phototherapy, and immune stimulation. Particularly for hormone receptor-positive (luminal) breast cancers, nanomedicine-enhanced apoptosis has great potential for overcoming acquired resistance to standard therapies. Notably, approaches based on immunogenic cell death (ICD) represent promising strategies for mitigating recurrence and metastasis in refractory triple-negative breast cancer subtypes. Future research should prioritize the optimization of nanoplatform composition and delivery efficiency by maximizing the remodeling of the tumor immune microenvironment and apoptosis induction to facilitate therapeutic efficacy and prevent relapse.

### 3.3. Mechanism of Nanoparticle-Induced Necroptosis

Unlike the classic receptor–ligand binding mode, the induction of necroptosis by nanomaterials seems to rely more on their inherent physicochemical properties that trigger this process by activating the RIPK1–RIPK3–MLKL signaling axis. Once inside cells, these nanoparticles disrupt energy metabolism and redox homeostasis, leading to the rapid accumulation of reactive oxygen species. When metabolic disorders and oxidative stress act in concert, MLKL is ultimately activated and perforates the cell membrane, destroying membrane integrity and thereby initiating immunogenic cell death [35,86].

Nanotechnology has offered novel strategies for the induction of necroptosis. Nanoparticles facilitate the enhancement of drug targeting precision and bioavailability; under optimized design parameters, they can elicit necroptosis in tumor cells, presenting a promising avenue for treating apoptosis-resistant malignancies [86]. By inducing immunogenic cell death (ICD), these nanoplatforms prime host immune responses, facilitate the circumvention of chemotherapy resistance, and increase the efficacy of cancer immunotherapy [87].

Nanoparticles have been demonstrated to stimulate necroptosis in breast cancer cells through various signaling pathways, among which the RIPK1–RIPK3–MLKL axis has emerged as a primary signaling axis [35]. Mechanistically, following intracellular transport, nanoparticles activate upstream sensors such as TNF receptor 1 (TNFR1/Fas), TLR3/4, or the nucleic acid receptor Z-DNA-binding protein 1 (ZBP1). These signals trigger the recruitment of RIPK1, which forms the necrosome by binding with RIPK3 via the RHIM domain. Activated RIPK3 subsequently phosphorylates MLKL, precipitating MLKL oligomerization, membrane insertion, and pore formation, which ultimately leads to the loss of membrane integrity, cell rupture, and DAMP release. Caspase-8 negatively regulates this pathway by cleaving RIPK1 or RIPK3; thus, necroptosis is predominantly favored under conditions of caspase-8 inhibition or the absence of cellular inhibitors of apoptosis proteins (cIAPs) [35]. Concurrently, nanoparticles may further promote necroptosis and enhance antitumor properties by modulating oxidative stress [35].

The RIPK1, RIPK3, and MLKL proteins constitute the core signaling axis in necroptosis. In breast cancer cells undergoing nanoparticle-mediated necroptosis, this axis is frequently robustly activated. Specifically, in triple-negative breast cancer models, silver-chitosan nanoparticles with incorporated shikonin exert synergistic effects in upregulating RIPK3 expression while concurrently increasing the phosphorylation of both RIPK3 and MLKL, thereby initiating necroptosis. This event is characterized by concomitant immunogenic cell death (ICD) and enhanced antitumor immune responses. In this study, this induction of necroptosis was rigorously validated through both pharmacological inhibition and genetic approaches, which confirmed the essential role of the RIPK3–MLKL axis in nanoparticle-mediated cell death [66]. Upon the codelivery of MET and DOX via FCA nanocarriers, tumor cells exhibited elevated MLKL expression, along with the simultaneous activation of the pyroptosis effector GSDMD and the apoptosis-related protease caspase-7. Collectively, these alterations indicate the occurrence of PANoptosis, a coordinated engagement of pyroptosis, apoptosis, and necroptosis [88]. Critically, the crosstalk among these pathways is not a coincidental overlap but rather a highly coordinated network rooted in shared regulatory hubs, such as those involved in redox homeostasis. For instance, nanoparticle-mediated depletion of intracellular glutathione disables the redox buffering capacity of cells. This disruption simultaneously induces lipid peroxidation to drive ferroptosis [89] and amplifies oxidative stress to activate the multimeric PANoptosome scaffold [90]. Such engineered crosstalk enables nanomedicines to engage compensatory death mechanisms. Consequently, if a highly heterogeneous cancer subclone silences classical apoptotic cascades to evade therapy, the death signal is executed via alternative inflammatory modalities, such as MLKL-dependent necroptosis or GSDMD-dependent pyroptosis. Ultimately, engaging these interconnected nodes effectively eliminates compensatory escape routes, offering a robust strategy to overcome multidrug resistance. Notably, this mechanism may be particularly applicable to heterogeneous breast cancers, where subclonal diversity frequently drives therapeutic evasion [91]. Similarly, PLGA nanoparticles loaded with *Brassica napus* extract (BNE-PNPs) can induce programmed necrosis in human MCF-7 breast cancer cells (a representative luminal A subtype model), accompanied by the upregulated expression of necroptosis-related genes such as tumor necrosis factor-α (*TNF-α*) and *p53* [92]. In addition, PLGA nanoparticles loaded with docetaxel (PLGA-Dtx) increased RIPK1 and RIPK3 expression and promoted necroptosis in a non-small cell lung cancer model, supporting the ability of drug-loaded nanosystems to modulate the RIPK1–RIPK3–MLKL axis [93]. mRNA-protective nanocage technology has also been utilized for the delivery of *RIPK3* mRNA to increase its expression at tumor sites, which further highlights the pivotal role of RIPK3 in nanoparticle-induced necroptosis [94]. Taken together, these findings suggest that the activation of the RIPK1–RIPK3–MLKL axis is central to nanoparticle-induced necroptosis in breast cancer, a process that is frequently integrated with inflammatory and immune activation.

**Figure 3:**
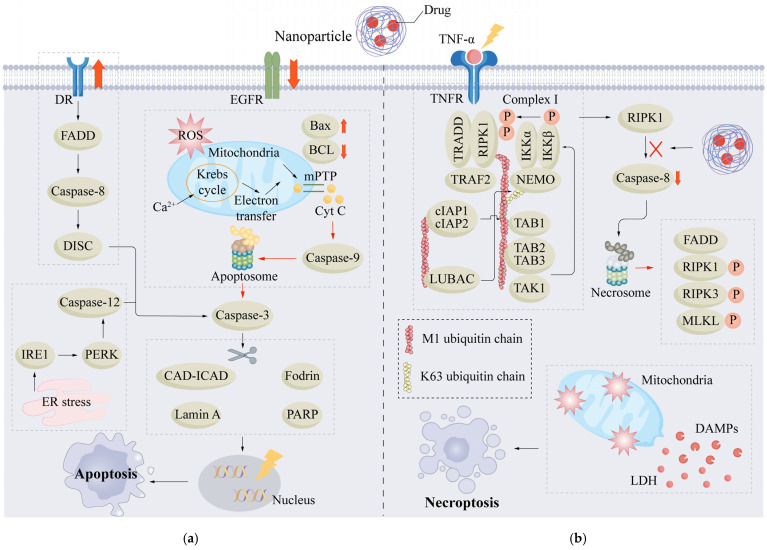
The dual programmed mechanisms of nanoparticle-induced cell death in breast cancer cells: apoptosis and necroptosis. (**a**) Apoptosis mechanism: After nanoparticles enter the cells through endocytosis, they mainly activate three parallel and crosstalk existing classical apoptotic pathways: (1) Mitochondrial (intrinsic) pathway: triggered by reactive oxygen species (ROS) or DNA damage, resulting in the release of cytochrome C and the formation of apoptotic bodies, activating Caspase-9; (2) Death receptor (extrinsic) pathway: formed by ligand-receptor interaction, activating Caspase-8; (3) Endoplasmic reticulum stress pathway. These pathways eventually converge on the executor Caspase-3/7 for activation, cleaving key substrates, and causing DNA fragmentation and nuclear condensation, thereby clearing the cells in a highly ordered manner. (**b**) Necroptosis mechanism: When nanoparticles inhibit the key executors of apoptosis (such as Caspase-8), the cell fate will shift to programmed necrosis. After the initiation of signals such as tumor necrosis factor-alpha/tumor necrosis factor receptor 1 (TNF-α/TNFR1), the inhibited apoptotic pathways cause receptor-interacting protein kinase 1 (RIPK1) to remain continuously activated due to its inability to be cleaved. Activated RIPK1 recruits and phosphorylates receptor-interacting protein kinase 3 (RIPK3), forming a necrosome. Subsequently, RIPK3 phosphorylates the terminal effector protein mixed lineage kinase domain-like protein (MLKL), leading to its oligomerization and translocation to the cell membrane to form channels, disrupting membrane integrity and triggering cell swelling, content release, and inflammatory death. Upward-pointing orange arrows represent the up-regulation of pro-apoptotic factors (e.g., Bax), while downward-pointing orange arrows denote the downregulation of anti-apoptotic proteins (e.g., Bcl-2). Thin red arrows highlight the key underlying mechanisms and critical regulatory steps. Black arrows indicate biological processes. Red cross signs (×) indicate the blockage or inhibition of specific signaling cascades by the therapeutic agents.

Nanoparticles not only can directly initiate necroptotic signaling but may also indirectly facilitate it by altering cellular energy metabolism and redox homeostasis. Evidence indicates that nanoparticles can significantly increase the level of intracellular ROS and that high levels of ROS can facilitate the potentiation of necroptotic signaling. Specifically, FePdNZ can be incorporated into functional lipid nanoparticles (FPS-LNPs) by its combination with shikonin. In this system, nanozyme activity results in robust generation of ROS, which augments the shikonin-induced necroptosis activity, exerts a potent inhibitory effect on tumor growth, and activates host immune responses [95]. Nanoparticle-induced ROS not only can potentiate the process of necroptosis but may also engage in crosstalk with the activity of other types of programmed regulated cell death (RCD) to yield superior antitumor effects. For example, shikonin, when combined with Fe^3+^, can form Fe(III)-shikonin nanoparticles (FSSNs), which release Fe^3+^ intracellularly and elicit necroptosis through Fenton-type processes; this mechanism potentially synergizes with other regulated cell death (RCD) mechanisms to increase antitumor efficacy [60]. Similarly, nanoparticles of magnesium-doped piezoelectric hydroxyapatite (MHMO) can simultaneously liberate Mg^2+^ ions and ROS/Ca^2+^ when stimulated by ultrasound. The combination of inflammation induced by necroapoptosis and TCR conformational remodeling mediated by Mg^2+^ can activate the NF-κB pathway, resulting in macrophage polarization toward the M1 phenotype. Concurrently, the signaling of ROS/Ca^2+^ also increases the expression of death receptor 5 (DR5), necroapoptosis in tumor cells, and immunogenic cell death and immunogenic activity in the microenvironment [96]. Taken together, these findings suggest that nanoparticles can potentiate necroptotic signaling by increasing ROS levels and integrate other modes of Regulated Cell Death and immunogenic cell death to orchestrate multimodal antitumor activity. However, future research should focus on developing stimuli-responsive nanosystems that precisely modulate ROS levels to regulate necroptosis and elucidate interactions with other modes of Regulated Cell Death and the immune response. These developments could increase the efficacy of cancer immunotherapy and circumvent drug resistance in tumors more efficiently (Figure 3).

## 4. Autophagy

### 4.1. Biological Characteristics of Autophagy and Its Dual Roles in Breast Cancer

The self-degradation process known as autophagy is an evolutionarily preserved mechanism that degrades defective cellular organelles and abnormal proteins through the autophagosome–lysosome pathway, thus sustaining cellular homeostasis and energy metabolism [97]. Autophagy exhibits marked, context-dependent dualism in breast cancer. Under physiological conditions, autophagy facilitates mitigation of the development of tumors by clearing damaged cellular components. Conversely, when tumor microenvironment stressors (nutrient deprivation, hypoxia, or therapy-induced stress) are present, autophagy may transition to a cytoprotective mode to promote cancer cell survival, drug resistance, and metastasis [97,98,99]. Because breast cancer cells develop an addiction to TME-driven autophagic adaptation to survive chronic hypoxia and nutrient scarcity, they exhibit marked vulnerability to targeted autophagic interference. Nanoparticles strategically exploit this dependence by either blocking protective autophagic flux or inducing autophagy-dependent immunogenic cell death, thereby disrupting the metabolic resilience of tumors within the hostile microenvironment and attenuating the barrier functions of cancer associated fibroblasts [100]. Consequently, current therapeutic strategies are generally categorized into two distinct types: suppression of cytoprotective autophagy to increase therapeutic efficacy and stimulation of cytotoxic (lethal) autophagy to eradicate tumor cells directly.

### 4.2. Mechanisms of Nanoparticle-Induced Autophagy in Breast Cancer Cells

For the regulation of autophagy, nanoparticles function by inhibiting the mTOR signaling pathway, inducing excessive production of reactive oxygen species, and modulating the expression of specific autophagy-related genes. These combined mechanisms disrupt cytoprotective homeostasis within tumor cells, ultimately converting pro-survival signals into lethal autophagic cell death [97,101].

Autophagy is frequently exploited by tumor cells to resist treatment pressure. Studies have demonstrated that the chemoresistance of triple-negative breast cancers is closely associated with autophagy activation, but inhibition of autophagy can significantly increase chemosensitivity. It should be mentioned that this causal relationship was rigorously validated through the use of *LC3* siRNA-mediated genetic knockdown, which explicitly confirmed the functional role of autophagy in treatment resistance [102]. Similarly, cytoprotective autophagy induced by photothermal therapy (PTT) and photodynamic therapy (PDT) may be detrimental to therapeutic efficacy. The solution to this limitation is the Tf-Te/HCQ system, which uses transferrin (Tf) to specifically recognize tumor cells and hydroxychloroquine (HCQ) to alkalinize lysosomes. It also inhibits autophagic flux, along with Fe^2+^-mediated lysosomal injury, resulting in the accumulation of LC3-II and p62 and hence sensitizing tumors to phototherapy [103]. Additionally, autophagy has been demonstrated to maintain the stemness of cancer stem cells (CSCs) and establish an immunosuppressive environment, which facilitates immune evasion and promotes disease progression [101,104]. Building upon these findings, there are nanoplatforms such as reduction-sensitive RNA interference mechanisms that can suppress autophagy to alleviate cytoprotection in tumor cells and increase ROS stress to elicit immunogenic cell death (ICD) and potentiate antitumor immune responses [105].

Excessive autophagy activation can eliminate tumor cells in certain scenarios with direct death due to metabolic collapse or fatal signaling pathways. Specifically, stimulation of autophagy can increase endoplasmic reticulum (ER) stress, induce immunogenic cell death (ICD), increase the rate of SNAI1 decay to prevent epithelial–mesenchymal transition (EMT), and collectively impede tumor growth and metastasis [106]. *PTEN* loss can constitute autophagy activation as a critical juncture of immune priming in tumors. Lipid nanoparticles (PepLNPs) that target programmed death ligand 1 (PD-L1) elicit high levels of autophagic stress, which is mediated by the re-expression of PTEN and inhibition of the PI3K–AKT–mTOR pathway. This is associated with calreticulin (CRT) exposure and ATP and HMGB1 release, which promote dendritic cell (DC) maturation and CD8+ T-cell infiltration, ultimately resulting in the abrogation of immune tolerance and effective suppression of tumor progression and metastasis [107].

Autophagy is regulated in breast cancer cells by nanoparticles in several ways, primarily via the modulation of mammalian target of rapamycin (mTOR) signaling pathway and the expression of autophagy-related genes. mTOR has been identified as one of the main negative regulators of autophagy, and inhibiting mTOR signaling stimulates the initiation of autophagy [101,108]. Autophagy can be elicited by nanoparticles that modulate the AMPK pathway or by direct inhibition of the mTOR pathway. Rapamycin is an established mTOR inhibitor, and rapamycin-encapsulating nanosystems can effectively activate autophagy and increase the chemosensitivity of breast cancer cells [109]. Similarly, nanoparticle delivery systems encapsulating curcumin (CUR) inhibit the PI3K–Akt–mTOR pathway, thereby alleviating autophagy suppression and activating autophagy in breast cancer cells [110]. In addition, nanoparticles can modulate autophagy-related regulators such as Beclin-1 and LC3 at the transcriptional or translational level, thereby remodeling autophagic flux and disrupting the cytoprotective autophagy balance in tumor cells [111]. In the nano-CUR system, the delivery of CUR to breast cancer cells activates the ROS–AMPK–ULK1 axis and suppresses PI3K–Akt–mTOR signaling, promoting the dissociation of Bcl-2 from Beclin-1 and increasing ATG-mediated LC3-II formation, which potentiates autophagy. Autophagic surcharge subsequently downregulates the expression of breast cancer stem cell (BCSC) markers and antiapoptotic proteins, induces type II Regulated Cell Death, reduces Treg populations, and increases the activity of cytotoxic T lymphocytes and natural killer cells, thereby exerting synergistic autophagy–immune anticancer effects [110]. Independent of mTOR-mediated regulation, small-molecule complexes such as NMK-T-057 induce autophagic death in 4T1 cells (a widely used triple-negative breast cancer model) by targeting the γ-secretase complex, inhibiting the release of the Notch intracellular domain (NICD), and downregulating Hes1 expression [112]. In parallel, nanoplatforms such as N1-ABT-NPs block Notch1 receptors, sensitize cells to ABT-737, target Bcl-2 family proteins, and induce cell death in triple-negative breast cancer (TNBC) cells [112].

Nanoparticle-induced autophagy is also closely associated with ROS signaling. Constructed Ce6-MnO_2_-BSA (CMB) nanoparticles deplete GSH in the tumor Nanoparticle-induced autophagy is also closely associated with ROS signaling. Constructed Ce6-MnO_2_-BSA (CMB) nanoparticles deplete GSH in the tumor microenvironment (TME) and catalyze the decomposition of H_2_O_2_ to generate substantial quantities of hydroxyl radicals (·OH) and O_2_, resulting in ROS bursts. Excessive ROS serve as a critical signal to trigger pro-death autophagic flux, characterized by pronounced autophagosome accumulation, enhanced LC3-I to LC3-II conversion, and reduced p62 expression, indicating increased autophagic activity rather than flux blockade [113]. Numerous metal-based nanoparticles increase intracellular ROS levels and subsequently activate autophagic pathways through oxidative stress induction [114,115]. For example, gold nanoparticles loaded with Xanmaolian and adriamycin act on breast cancer stem cells (BCSCs) to target the transferrin receptor and trigger NCOA4-mediated ferritinophagy, resulting in elevated concentrations of Fe^2+^ and increased ROS production due to the Fenton reaction. This process blocks mTORC1, activates AMPK, releases ULK1 inhibition, and induces autophagy, thus creating a positive feedback loop between ferroptosis and autophagy and successfully eliminating breast cancer stem cells [116]. ROS-induced autophagy is an important adaptive response of tumor cells to microenvironmental stress, and its biological consequences are highly dose dependent. Excessive ROS accumulation is characterized by an overabundance of autophagosomes and autophagic cell death, and moderate ROS elevation has been shown to elicit cytoprotective autophagy via AMPK-associated signaling, facilitating tumor cells to resist the stress of therapy. Such bidirectional regulation forms the basis of the dual role of ROS-mediated autophagy in cancer treatment, which could be identified as an anti-ROS toxicity mechanism and cell viability maintenance along with a potential sensitization target [117].

Additionally, other nanoparticles can enhance biocompatibility through surface functionalization, specifically ligand conjugation or charge modulation, which facilitates the identification of breast cancer cells with greater specificity and precisely regulates the process of autophagy [118,119]. In conclusion, nanoparticles can activate autophagy in breast cancer cells by modulating mTOR signaling, inducing the production of ROS and modifying autophagy-associated gene expression. Drug resistance in breast cancer is anticipated to be circumvented by the precise modulation of autophagic processes by nanoparticles and emerge as a feasible treatment approach [97,120]. Future research needs to clarify how autophagy-inducing mechanisms vary among different nanoparticles and how they impact different breast cancer subtypes, which will provide a theoretical framework for the design of more specific and safer nanomedicines.

## 5. Ferroptosis

### 5.1. Biological Characteristics of Ferroptosis

Ferroptosis was first defined by Brent R. Stockwell in 2012 [67]. It is a regulated form of cell death driven by iron-dependent lipid peroxidation [68]. Its execution depends on an imbalance between lipid oxidative damage and cellular antioxidant defense systems [121,122,123]. Acyl-CoA synthetase long-chain family member 4 (ACSL4) and lysophosphatidylcholine acyltransferase 3 (LPCAT3) facilitate the incorporation of polyunsaturated fatty acids into membrane phospholipids, thereby supplying substrates for lipid peroxidation [124]. Intracellular iron catalyzes lipid peroxide generation through the Fenton reaction and lipoxygenase activity [68]. Cysteine uptake via the cystine/glutamate antiporter system supports glutathione synthesis and sustains GPX4 antioxidant activity [67,125]. In addition, ferroptosis inhibitory protein 1 (FSP1) cooperates with GPX4-independent pathways, such as those involving dihydroorotate dehydrogenase (DHODH), to preserve membrane lipid homeostasis [126,127,128]. When iron homeostasis is perturbed or GPX4 function is compromised, excessive accumulation of lipid peroxides disrupts membrane integrity and ultimately triggers ferroptotic cell death [69,129].

### 5.2. Mechanisms of Nanoparticle-Induced Ferroptosis in Breast Cancer Cells

Nanoparticles can augment the intracellular iron pool via the release of iron ions, thereby depleting glutathione and suppressing GPX4 expression. They facilitate extensive lipid peroxidation through the Fenton reaction. Certain nanoparticles additionally release iron via mechanical disruption of the lysosomal membrane. These concerted mechanisms collectively disrupt intracellular redox homeostasis [130,131].

Nanoparticles have shown significant potential to induce ferroptosis because of their favorable physicochemical and cellular targeting capabilities. Nanoparticles can facilitate the process of ferroptosis by modulating the level of intracellular iron homeostasis and ROS. However, the mechanisms underlying the induction of ferroptosis differ fundamentally between organic and inorganic nanoplatforms. Inorganic nanoparticles function as active biochemical participants by directly providing transition metal ions to increase the intracellular labile iron pool and catalyze lipid peroxidation via the Fenton reaction. In contrast, organic nanoparticles typically serve as multifunctional carrier platforms that do not directly catalyze reactive oxygen species generation; instead, they compromise cellular antioxidant defense systems by delivering specific effector molecules (e.g., GPX4 inhibitors or glutathione-depleting agents), thereby indirectly triggering ferroptosis [132,133,134]. In the TME context, breast cancer cells frequently increase GSH and antioxidant defense systems, which provides a formidable barrier against lipid peroxidation. Smart nanoparticles circumvent this resistance by actively depleting the aberrantly high GSH pool and exploiting endogenous H_2_O_2_ to fuel Fenton-like reactions. This active intervention disrupts redox homeostasis within the TME, effectively sensitizing breast cancer cells to ferroptotic cell death which further aids in repolarizing tumor associated macrophages toward an anti tumor phenotype [134].

However, the susceptibility to ferroptosis significantly varies among breast cancer subtypes [135]. TNBC cells in particular often overexpress the cystine/glutamate antiporter (SLC7A11) to manage high basal oxidative stress [136]. Consequently, they exhibit heightened dependency on GSH maintenance, creating a metabolic ‘Achilles heel’ that nanoparticles can exploit through targeted GSH depletion [135,137]. In contrast, luminal subtypes may exhibit relative resistance to ferroptosis because of decreased basal oxidative stress or alternative antioxidant pathways (e.g., the thioredoxin system), necessitating subtype-specific therapeutic strategies [138,139]. Iron-based nanoparticles can liberate Fe^2+^ ions, expand the intracellular labile iron pool, and catalyze lipid peroxidation. For example, ssP-tHBFe polymer micelles, which are enriched with disulfide bonds and loaded with high-valence Fe^3+^, have been demonstrated to promote ferroptosis [137]. In terms of the HER2-positive subtype, targeted nanoplatforms have distinct advantages; for example, the PPAPH nanoplatform can markedly elicit ferroptosis in HER2-positive breast cancer cells through synergistic chemo-photothermal therapy, primarily through increased ROS levels, disrupted lipid metabolism, and redox homeostasis imbalance [140]. Nanoparticles can further potentiate lipid peroxidation and ferroptosis by inhibiting GPX4 expression, depleting glutathione, and promoting ROS generation [130]. Functional iron-based nanoparticles (FHA NPs) constructed from hyaluronic acid and iron ions selectively accumulate in tumor cells via CD44-mediated endocytosis. These nanoparticles induce ROS generation and lipid peroxidation through the Fenton reaction, downregulate GPX4 expression, and trigger ferroptosis while being minimally cytotoxic toward normal cells. In this study, the induction of ferroptosis in this model was conclusively confirmed via rescue experiments using gold-standard inhibitors and genetic validation, providing robust causal evidence for the reported mechanism [131]. Ferroptosis induced by gold nanoparticles (AuNPs) is characterized by intracellular iron overload, GSH depletion, reduced GPX4 activity, ROS accumulation, and lipid peroxidation, culminating in mitochondrial dysfunction and membrane rupture [141]. Cu-Pb nanoparticles attenuate GPX4 antioxidant activity through a combination of synergistic depletion of intracellular GSH and sustained elevation in ROS levels and thus promote the accumulation of lipid peroxides until the threshold of membrane integrity loss and robust activation of ferroptosis in triple-negative breast cancer (TNBC) [142]. IP-ss-FRT is a nanoparticle system derived from ferritin that, in response to heat and hyperthermia as well as the effects of glutathione, precipitates the release of Fe^2+^ and facilitates ferroptosis upon photothermal therapy (PTT). This system results in high levels of intracellular GSH depletion and weakened antioxidant defenses and facilitates the efficient eradication of drug-resistant breast cancer cells [143]. Additionally, new studies have extended the potential applications of nanoparticles, showing that nanoparticles can not only trigger ferroptosis in tumor cells but also prevent the death of immune cells due to ferroptosis. For example, nanoparticles coated with human serum albumin (LHS NPs) specifically induce ferroptosis in tumor cells and suppress ferroptosis in CD8+ T cells, thus increasing the antitumor effects of the immune system and circumventing the limitations of conventional treatments, causing immunosuppression in patients with tumors [144].

Nanoparticles can also increase ferroptosis by increasing lysosomal membrane permeability (LMP). NBTXR3 nanoparticles induce lysosomal membrane permeabilization in tumor cells, promote lipid peroxide accumulation, accelerate ferroptosis, and enhance radiotherapy efficacy upon radiation activation [145]. In recent years, nanomaterial-derived mechanical forces have been utilized to regulate LMP and amplify ferroptosis [146]. Studies have shown that T7 peptide-modified magnetic nanoparticles (T7-MNTs) target transferrin receptors on breast cancer cells and accumulate within lysosomes. Under a low-frequency rotating magnetic field, they assemble into rod-like clusters that generate ~36.4 pN of torque, mechanically disrupting lysosomal membranes and triggering massive Fe^2+^ release and lipid peroxidation. This process converts lysosomes into ferroptosis-initiating platforms and provides a low-toxicity, long-acting, and programmable physicochemical sensitization strategy for deep-seated tumors [146]. In addition, studies in TNBC (a highly aggressive TNBC model) have demonstrated that ionizing radiation can induce a nonapoptotic, nonclassical ferroptotic form of cell death. Ionizing radiation increases lysosomal membrane permeability, leading to iron leakage from lysosomes. Released iron reacts with cytosolic H_2_O_2_ to generate hydroxyl radicals and trigger ROS bursts. Increased ROS levels are accompanied by autophagy activation, which promotes ferritin degradation and further iron release, continuously amplifying oxidative stress and ultimately driving cell death [147]. Owing to this nonclassical death mechanism, several important questions arise. How should lysosome-specific nanoparticles be engineered to specifically increase LMP-induced iron release upon irradiation with ionizing radiation? What is the methodology for quantifying the ROS level needed to engage autophagy? How to predict that TNBC may be sensitive to both ionizing radiation and nanoparticle therapies? Moreover, research in colorectal cancer has also indicated that one lysosome-targeted photosensitive agent (TLA) can induce lysosomal lysis and inhibit autophagy under photodynamic therapy to further potentiate ferroptosis, initiate immunogenic cell death, remodel the immune microenvironment, and combine with PD-L1 antibodies to eradicate drug-refractory malignancies [148]. Similarly, colorectal cancer shares characteristics with TNBC, as it is characterized as an immune-cold type of tumor, and the mono-therapeutic use of immune checkpoint inhibitors does not result in substantial clinical efficacy [149,150]. These findings suggest that combination therapy with ferroptosis-inducing strategies directed at lysosomes plus immunotherapy might be used to treat breast cancer. The most significant unsolved question is whether the breast cancer molecular subtypes vary in their lysosomal iron storage capacities and ferroptosis sensitivities.

Collectively, these findings indicate that ferroptosis is primarily driven by iron-induced lipid oxidation. Nanoparticles may significantly increase ferroptosis through the regulation of intracellular iron accumulation, as well as through reactive oxygen species and antioxidant defense mechanisms. Specifically, for the HER2-overexpressing subtype, the use of targeted nanocarriers to induce specific lipid metabolism collapse and redox imbalance represents a highly promising translational strategy.

Importantly, the susceptibility of breast cancer cells to nanoparticle-induced ferroptosis is deeply intertwined with their metabolic reprogramming [134,151], and the robust glutamine metabolism and heightened de novo lipogenesis required for rapid tumor proliferation inherently create a PUFA-enriched and high-ROS environment, rendering the disruption of redox and iron homeostasis a lethal metabolic vulnerability upon targeted interference [139,152]. Future detailed research on nanoparticle-induced ferroptosis mechanisms will aid in the development of new strategies and provide a robust theoretical foundation for the precision therapy of breast cancer and other types of cancers.

## 6. Cuproptosis

### 6.1. Biological Characteristics of Cuproptosis and Its Roles in Tumor Cells

Cuproptosis is a novel cell death mechanism that was characterized in 2022. It consists of copper ionophores that introduce Cu^2+^ into mitochondria, where ferric redox protein (FDX1) reduces Cu^2+^ to Cu^+^ and facilitates copper binding to oligomerizing lipoylated tricarboxylic acid (TCA) cycle enzymes. As a result of this process, iron–sulfur cluster biogenesis is disrupted, proteotoxic stress is induced, and ultimately, cell death ensues [153]. Given that aggressive breast cancer cells still fundamentally rely on mitochondrial oxidative phosphorylation (OXPHOS) for metabolic plasticity and macromolecule synthesis [154], this copper-induced aggregation of lipoylated TCA cycle enzymes acts as a targeted metabolic poison, directly paralyzing the mitochondrial hub and precipitating an irreversible bioenergetic crisis [153]. Notably cuproptosis may be reversed by copper chelators and exhibits crosstalk with ferroptosis and apoptosis, pyroptosis and other regulated cell death processes. These interactions suggest that cuproptosis has the potential to not only initiate tumor cell death but also trigger antitumor immune responses, making it a promising therapeutic approach for cancer treatment [155]. High YAP expression has been identified as a primary determinant of cuproptosis susceptibility [156]. High YAP levels are frequently associated with aggressive features of TNBC, in which they upregulate the expression of copper-binding proteins such as ATOX1. This molecular signature suggests that cuproptosis-inducing nanoparticles may be particularly effective for these high-YAP-expressing cohorts, offering a potential companion diagnostic marker for clinical stratification [156].

The potentiating effect of ferroptosis on cuproptosis is synergistic. Copper ion retention due to the administration of T-TCu attenuates ATP synthesis during ferroptosis induction, inhibits the activity of ATP7A/ATP7B indirectly, and facilitates long-term intracellular copper sequestration. The accumulated copper ions catalyze hydroxyl radical (·OH) production through Fenton-type oxidation and increase ROS production and lipid peroxide accumulation, which synergistically potentiate downregulation of GPX4 expression. This precipitates a cascade of excessive copper load, depleted ATP, and ROS amplification that ultimately leads to increased tumor cell lethality [157]. This synergy involves the precise regulation of intracellular levels of copper and its association with metabolic enzymes, thus making it a potential vulnerability for therapeutic targeting in tumor cells.

### 6.2. Mechanisms of Nanoparticle-Induced Cuproptosis in Breast Cancer Cells

Nanoparticles induce copper-induced cell death through specific metabolic pathways. By functioning as targeted delivery vehicles, they increase intracellular copper accumulation, which subsequently depletes glutathione to compromise tumor defense mechanisms. Furthermore, nanoparticles catalyze the generation of reactive oxygen species via hydrogen peroxide decomposition. Collectively, these actions promote the lethal aggregation of lipidated proteins, thereby disrupting the tricarboxylic acid cycle and ultimately leading to cell death [158,159].

The regulation of cuproptosis is also significantly mediated by nanoparticles. Inorganic nanoparticles based on copper can serve as direct donors rather than mere carriers of copper ions, which selectively penetrate tumor tissue, increase the intracellular accumulation of copper, and subsequently induce cuproptosis. In particular, inorganic copper-based nanoplatforms such as CuS and CuO-2 have been shown to release copper ions in the tumor microenvironment, promote the aggregation of lipoylated proteins and the depletion of GSH, increase oxidative stress and ultimately induce cell death [158,159]. Cu(I)-BSA monoatomic nanozymes use a catalytic process to convert H_2_O_2_ into reactive oxygen species, reduce glutathione, lower ATP7A levels, and maintain Cu(I) within cells without oxidation into paramagnetic Cu(II) in tumors. Together, these processes initiate cuproptosis and intensify T-weighted MRI signals, enabling self-validating theranostics [160]. The hyaluronate-modified CuO-DOX clusters containing Cu^2+^ and H_2_O_2_ are internalized into the cell, liberating Cu^2+^ and converting H_2_O into ·OH and O_2_, increasing oxidative stress and alleviating hypoxia. DOX also potentiates the formation of endogenous H_2_O_2_ and increases the levels of intracellular ROS. The disulfide bonds in the carrier synergize with Cu^2+^ to deplete GSH, increase copper and ROS toxicity, and act synergistically to elicit cuproptosis [159]. Copper ions are released together with hydrogen peroxide by Cu-ZnO PDA in acidic conditions, resulting in oxidative stress and mitochondrial injury. Activation of the cGAS–STING pathway leads to dendritic cell maturation and T-cell infiltration, augments antitumor immunity, increases the expression of PD-L1, transforms cold tumors into hot tumors, and potentiates immune checkpoint inhibitor therapy [161]. Cuproptosis can therefore also be identified as a mechanism underlying the interaction between cellular metabolic activity and antitumor immune responses. Tumor targeting can be facilitated by the use of platelet membrane-coated PCB nanoparticles, with CuP releasing copper and inducing H_2_O_2_ catalysis to produce free radicals. The binding of dihydrolipoamide acetyltransferase (DLAT) to copper ions is mediated by FDX1 to form protein aggregates, which disrupt the tricarboxylic acid cycle and induce cuproptosis. Moreover, this system suppresses GSH levels, potentiates cuproptosis, and elicits antitumor immune responses, a mechanism further substantiated by molecular or genetic validation to confirm the specificity of copper-dependent cell death [162].

Notably, the induction of cuproptosis is not without limitations. Copper homeostasis can be maintained in tumor cells through upregulation of the expression of copper efflux transporters that attenuate the efficacy of induction of cuproptosis [163]. However, nanoparticles can enhance cuproptosis by inhibiting those efflux proteins and thus circumvent tumor tolerance and improve therapeutic outcomes [158], especially after eliciting ICD and activating antitumor immunity [163]. The CussOMEp nanoplatform impedes primary tumor progression, facilitates the maturation of DCs and promotes CD4^+^ and CD8+ T-cell infiltration. In combination with a PD-1 antibody, it exhibits a superior efficacy in suppressing remote tumors and lung metastasis and eliciting systemic antitumor immunity [163]. Although high GSH concentrations and hypoxia in the tumor microenvironment profoundly attenuate cuproptosis, CussOMEp generates ·OH, depletes GSH, relieves hypoxia and, through loading of the ATP7A inhibitor omeprazole, inhibits copper efflux, leading to robust accumulation of copper intracellularly [163].

As a result of further research developments, the application of nanoparticles has facilitated the integration of various therapeutic approaches. One example is where synergy among photothermal therapy (PTT), chemodynamic therapy (CDT), photodynamic therapy (PDT), and immunotherapeutic therapy yields synergistic antitumor benefits. Multifunctional nanoplatforms based on copper and containing photosensitizers, enzymes or immunomodulators have been developed to concurrently induce cuproptosis and alternative cell death pathways, stimulate immune activation, and inhibit tumor growth and metastasis [164,165,166]. In particular, mild photothermal heating increases copper ion-mediated Fenton-like reactions, induces increased ROS formation and accelerates ferroptosis. Cuproptosis decreases ATP concentrations and represses copper efflux proteins, thus increasing the retention of copper and the induction of cuproptosis. This interaction relieves the restrictions on the copper loading of mitochondria because of GSH scavenger activity and efflux transporters [157]. During the PTT process, CuS nanoparticles readily convert light into heat and induce irreversible thermal damage to tumor cells. Moreover, this photothermal action combines synergistically with cuproptosis, immune microenvironment remodeling and blocking of the C5a–C5aR pathway to potentiate therapeutic efficacy in breast cancer [158]. PTT increases the localized temperature of Cu-ZnO PDA, potentiates the production of copper ions and H_2_O_2_, and stimulates ROS generation through the Fenton reaction, which induce mitochondrial destruction and the release of mtDNA. Once released, mtDNA interacts synergistically with zinc ions to trigger cGAS–STING pathway activation, maturation of dendritic cells and T-cell infiltration, and an increase in cuproptosis-related immune cascades [161]. Overall, this multimodal treatment approach is highly efficacious in overcoming the major drawbacks of single cuproptosis therapy, such as a lack of sufficient copper supply, hypoxia in tumors, and high levels of glutathione.

Biocompatibility and the ability to target tumors constitute critical determinants when nanoparticles are designed. Strategies involving surface modification, such as platelet membrane coating, tumor cell membrane camouflage or ligand functionalization, yield nanoparticles with long circulation times, targeted in vivo accumulation, increased accumulation of copper in tumors, and minimized nonspecific toxicity [167,168]. To implement this strategy, the PCB nanoplatform was combined with metabolism intervention and immune regulation. It exhibited active tumor enrichment following systemic administration via intravenous administration facilitated by a PM coating mediated by P-selectin recognition and CD44. In the tumor microenvironment, CuP released Cu^2+^ continuously at high H_2_O_2_ levels, which was reduced with FDX1 to Cu^+^, which then bound to lipoylated DLAT, causing aberrant aggregation, which disrupted the tricarboxylic acid cycle and ultimately led to cuproptosis [162]. In addition, leveraging tumor microenvironment-responsive cues, such as acidic pH and enzymatic activity, to achieve controlled copper ion release and activity regulation has emerged as a pivotal focus in nanoparticle design [159,169]. DHCC-CuTH is a hollow calcium carbonate-based nanodelivery system that releases copper ions and disulfiram under acidic tumor conditions and generates CuET complexes in situ. CuET inhibits the ubiquitin–proteasome system and induces ER stress and calcium redistribution. Copper ions further induce DLAT aggregation and GSH depletion, potentiate oxidative stress, induce mitochondrial damage, and activate multiple cell death pathways. This system achieves targeted delivery to CD44-high breast cancer cells through hyaluronic acid modification and exhibits potent antitumor activity in breast cancer models, demonstrating remarkable antitumor efficacy in these models [170].

Therefore, cuproptosis—defined as a copper-dependent form of Regulated Cell Death characterized by the aberrant aggregation of mitochondrial lipoylated proteins and the depletion of iron–sulfur cluster proteins—presents a novel therapeutic paradigm for the treatment of breast cancer [155]. Nanosystems can reprogram copper metabolism, amplify death-related enzymatic activity, and cooperate with multimodal interventions to achieve lesion-selective activation, thereby advancing cuproptosis from the proof-of-concept stage to the preclinical translational stage. However, multiple challenges remain before large-scale clinical translation can be realized. Given the narrow therapeutic window of copper ions, how can overdose-induced vascular copper toxicity, particularly in cardiac tissues, be avoided? How can safety thresholds be defined to prevent systemic inflammatory autophagy without compromising cuproptosis-mediated antitumor efficacy?

**Figure 4:**
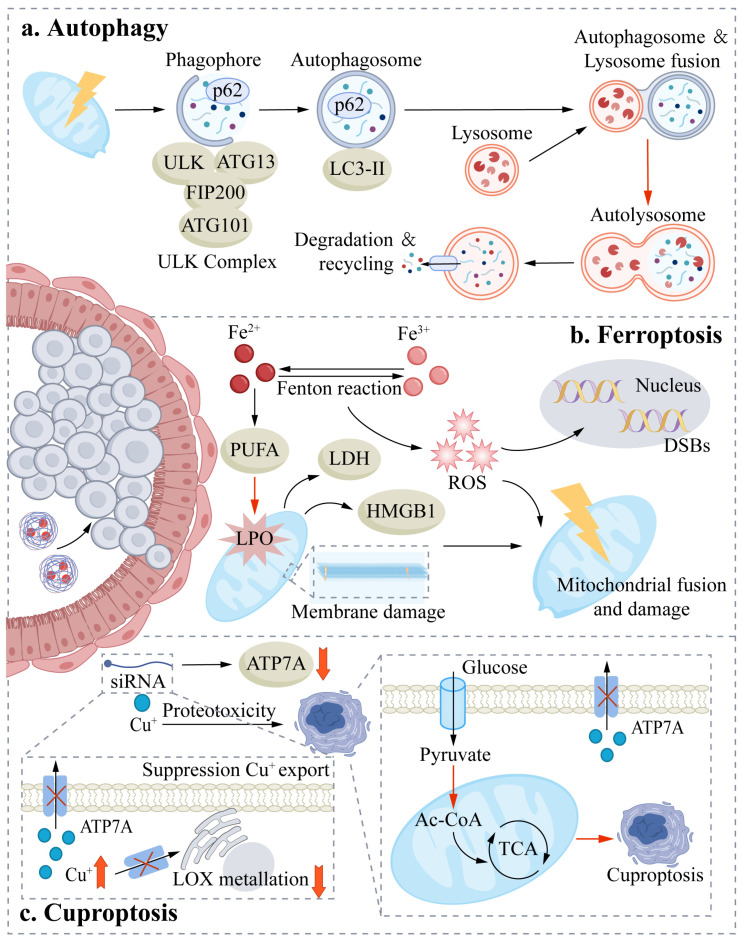
Schematic illustration of the mechanism by which nanoparticles treat breast cancer through induction of autophagy, ferroptosis, and copyloptosis. (**a**) Autophagy pathway: Nanoparticles can cause intracellular stress such as endoplasmic reticulum stress or mitochondrial damage, which in turn activates autophagy signaling. This pathway is initiated by ULK1 complex, Beclin-1 participates in the formation of autophagosomes, and through the key steps such as microtubule-associated protein 1 light chain 3 (LC3)-II lipidation, the damaged organelles are wrapped to form autophagosomes. Subsequently, autophagosomes fuse with lysosomes to form autolysosomes for degradation. Nanoparticle-induced excessive autophagy or disordered autophagic flow eventually leads to cell fate toward autophagy-dependent death. (**b**) Ferroptosis pathway: Iron ions (Fe^3+^) in autolysosomes produce ROS through Fenton reaction, which induces the peroxidation of polyunsaturated fatty acids (PUFA), leads to lipid peroxide (LPO) accumulation and membrane damage, and finally causes iron-dependent cell death. (**c**) Cuproptosis pathway: Nanoparticles interfere with the copper ion (Cu^+^) efflux protein adenosine triphosphatase copper transporting alpha (ATP7A), resulting in intracellular copper accumulation, inhibition of mitochondrial respiratory chain, proteotoxicity and abnormal mitochondrial fusion, thereby inducing copper-dependent cell death. Thin red arrows highlight the key underlying mechanisms and critical regulatory steps. Black arrows indicate biological processes. Red cross signs (×) designate inhibition, blockage, or pathway failure. Downward-pointing arrows indicating suppression or depletion, and upward-pointing arrows indicating up-regulation.

Therefore, although research on cuproptosis is rapidly expanding, successful clinical translation will require the construction of cuproptosis sensitivity prediction models based on multiomics data and AI, the development of subtype-selective nanodelivery systems, and the validation of combined cuproptosis–immunotherapy strategies in patient-derived organoid–microenvironment coculture models, ultimately enabling truly precise copper homeostasis-based therapy (Figure 4).

## 7. Disulfidptosis

### 7.1. Biological Characteristics of Disulfidptosis and Its Roles in Breast Cancer

Disulfide bonds are critical covalent linkages in proteins primarily formed through the oxidation of thiol groups from two cysteine residues, that thereby maintain protein conformational stability and functional integrity [171]. In breast cancer cells, disruption of disulfide bonds results in protein conformational alterations and functional loss, thereby triggering Regulated Cell Death [172]. Specifically, alterations in the intracellular redox state are key determinants of disulfide bond stability [173]. Nanoparticles can effectively mediate disulfide bond disruption by modulating the intracellular redox environment, thereby inducing cancer cell death [174].

### 7.2. Mechanisms of Nanoparticle-Induced Disulfidptosis in Breast Cancer Cells

Nanoparticles deplete intracellular glutathione to alter redox homeostasis while concurrently inhibiting glucose uptake, thereby inducing NADPH exhaustion and facilitating disulfide bond formation. This metabolic dysregulation triggers severe disulfide stress, which promotes aberrant actin crosslinking and cytoskeletal disintegration, ultimately suppressing tumor metastasis [175,176].

Disulfide bonds present in nanoparticles can function as reduction-sensitive linkers because when exposed to the highly reducing intracellular microenvironment of a breast cancer cell, bond cleavage triggers the release of therapeutic payloads, eliciting a cytotoxic response. The highly reducing nature of the TME enables responsive nanoparticles to achieve precise spatial–temporal control over drug release. Ultimately, these nanoplatforms translate the specific physicochemical cues of the TME into lethal ROS imbalance and severe disulfide stress, achieving highly specific tumor eradication while sparing normal tissues [173,177]. Paclitaxel prodrug nanoparticles incorporating glutathione-responsive disulfide linkages are cleaved by elevated concentrations of intracellular GSH, which releases paclitaxel along with the antimetastatic agent 30-hydroxypterostilbene (30-HPT). This release elicits drug-specific cytotoxic effects in breast cancer cells and effectively suppresses tumor metastasis by regulating metastasis-related protein expression and inhibiting epithelial–mesenchymal transition [178]. Similarly, paclitaxel can be linked to maleimide via disulfide bonds to construct prodrug nanoparticles (PSSMALs). After entering the bloodstream, PSSMALs bind to albumin and accumulate at tumor sites, where disulfide bond cleavage in the reducing environment releases active paclitaxel and induces cell death [179]. Similarly, IrssQu nanoparticles use disulfide bonds as reduction-sensitive bridges that are cleaved in the highly reducing intracellular environment, triggering irinotecan release from quinine, inhibiting P-glycoprotein (Pgp)-mediated efflux, increasing cytotoxicity, and reversing drug resistance [180]. In addition, SN38 (7-ethyl-10-hydroxycamptothecin) was linked via disulfide bonds to form d-SN38, which was further coassembled into d-SN38NPs with the photosensitizer Ce6 or BR-FFVLK-PEG complexes. Assisted by iRGD peptides, these nanoparticles efficiently accumulated in and penetrated tumors and were subsequently internalized by 4T1 cells (a widely used triple-negative breast cancer model). Elevated levels of intracellular GSH reduce and cleave disulfide bonds, leading to SN38 release, topoisomerase I inhibition, and apoptosis induction [181].

Nanoparticles can increase intracellular oxidative stress by depleting GSH or promoting ROS generation, thereby disrupting the redox balance between disulfide bonds and sulfhydryl groups and affecting protein disulfide bond formation and cleavage. Leveraging the highly reducing, GSH-rich environment of tumor cells, nanoparticles induce disulfide bond scission and the release of active drugs and, at the same time, deplete GSH and consequently increase oxidative stress [177]. Disulfide bond-containing metal–organic nanoparticles or gold nanocarriers used as drug-loading systems are capable of targeted drug delivery upon disulfide bond cleavage by GSH, which increases ROS production in tumor cells and ultimately leads to cell death [182,183]. In combination with nanoparticles, photodynamic therapy generates ROS through laser irradiation by Ce6-loaded nanocarriers, with Ce6 acting as a photosensitizer, activating the oxidative stress response synergistically with SN38 chemotherapy and exacerbating damage to tumor cells [181]. PEI-SS-VES nanoparticles are constructed by conjugating polyethylene imine (PEI) to vitamin E succinate through disulfide bonds that allow them to target both CD44 and αvβ3 receptors. They are cleaved and released into the GSH-rich tumor milieu as plasmids. Activation of GAVPO with blue light initiates diphtheria toxin A chain (DTA) expression, which inhibits eukaryotic elongation factor 2 (EF2) activity, which prevents the synthesis of proteins that lead to apoptosis in 4T1 cells (a widely used triple-negative breast cancer model) longation factor 2 (EF2), blocks protein synthesis, and induces apoptosis in 4T1 cells (a widely used triple-negative breast cancer model) [184].

Disruption of disulfide bonds affects not only the release of drugs but also the structure and function of pivotal proteins. The protein disulfide isomerase (PDI) family members ERp44 and AGR2 are key enzymes involved in the regulation of protein disulfide bond formation and rearrangement. Their inhibition or dysregulation leads to protein misfolding and facilitates the oligomerization of death receptors 4 and 5 through disulfide bonds, triggering apoptotic signaling pathways [185]. This mechanism implies that nanoparticles might indirectly elicit the eradication of breast cancer cells by modifying the activity of disulfide bond-related enzymes.

Disulfidptosis is a novel form of regulated cell death characterized by the aberrant accumulation of intracellular disulfide bonds, resulting in abnormal disulfide cross-linking of actin cytoskeletal proteins, cytoskeleton collapse and, subsequently, the inhibition of tumor cell migration and metastasis [175]. Following this approach, nanoparticle preparations incorporating disulfide bonds were developed to induce intracellular disulfide stress and demonstrated considerable antimetastatic properties [186]. Disulfide stress may also synergize with various regulated cell death pathways to maximize anticancer effects. The composition of CCDRF includes celastrol (Cel), dihydroartemisinin (DHA), and copper cations. With this nanoplatform, a marked reduction in the expression of glucose transporter (GLUT1), the concentration of intracellular glucose, the level of NADPH, a pronounced increase in the amount of cystine, and the constriction of F-actin were observed. Interestingly, this research revealed that the mechanism of lethality was twofold, consisting of disulfidptosis and cuproptosis. Cel downregulated GSH synthase expression through the inhibition of nuclear factor-κB (NF-κB), thereby inhibiting GSH, and intracellular Cu^2+^ was reduced by GSH, thus exhausting GSH and potentiating cuproptosis. The loss of reducing capacity precipitated increased disulfide stress, which facilitated increased damage to F-actin cross-linking. Therefore, the combination of GSH depletion due to Cel-Cu-mediated metabolic blockade with GSH depletion due to DHA-induced metabolic blockade ensured rapid tumor cell lethality through two distinct mechanisms, namely, cytoskeletal disruption and disruption of the GSH redox system [176]. FeOOHFe-ApAuNSs trigger both disulfide stress-induced lethality and ferroptosis in the tumor microenvironment. AuNDs mimic the activity of glucose oxidase to promote excessive downstream glucose uptake and substantially inhibit the production of NADPH and the conversion of cystine into cysteine mediated by SLC7A11. Exorbitant accumulation of cystine triggers actin disulfide cross-linking and initiates disulfidptosis. Simultaneously, the deficiency in cysteine and GSH downregulates expression of GPX4, which compromises the process of lipid peroxide elimination. This process further liberates Fe^2+^ and furnishes endogenous hydrogen peroxide, thereby accelerating the kinetics of Fenton-type reactive oxygen species (ROS) formation, which ultimately precipitates the induction of ferroptosis [187]. This study was conducted utilizing an ovarian cancer model; however, a systematic glucose deprivation strategy can be extrapolated to breast cancer, especially a TNBC model. TNBC cells exhibit robust expression of GLUT1 [188] and SLC7A11 [189], utilize the pentose phosphate pathway (PPP) as a substrate for NADPH production [190], and are highly sensitive to the processes of GSH synthesis [191]. These unique subtype-specific metabolic vulnerabilities render TNBC cells inherently more susceptible to disulfidptosis induced by targeted nanomaterials. A key clinical question is whether synergistic nanoplatforms, such as FeOOHFe-ApAuNSs, can induce comparable glucose deprivation in TNBC by targeting GLUT1 and promoting GOx-like consumption. As a functional implementation of this strategy, the biomimetic nanoplatform CYBC NPs utilizes the GLUT1 inhibitor BAY-876 and a homologous tumor membrane coating to increase targeting. By exploiting the metabolic heterogeneity of TNBC, CYBC NPs induce intense disulfide stress through glucose uptake inhibition and exogenous cystine supply, triggering irreversible F-actin cytoskeletal collapse. This subtype-stratified approach not only eradicates tumor cells directly but also initiates immunogenic cell death. The subsequent release of damage-associated molecular patterns promotes M1 macrophage polarization and activates CD4^+^ and CD8^+^T cells, thereby restructuring the immunosuppressive microenvironment and establishing long-term immune memory. Notably, this metabolic cell death pathway has been rigorously validated using both pharmacological inhibitors and genetic tools, unequivocally confirming the induction of disulfidptosis and the subsequent remodeling of the tumor microenvironment. This transforms disulfidptosis from a theoretical concept into a precision therapeutic strategy for the most refractory forms of breast cancer [192].

In summary, nanoparticles may induce different types of regulated cell death, such as apoptosis, immunogenic cell death, and the recently discovered disulfide stress-mediated cell death, by altering the intracellular redox status, inducing disulfide bond disruption and modulating protein structure and activity. These pathways offer a key strategy and theoretical basis underlying specific therapeutic strategies for treating breast cancer. Synergistic mechanisms of cell death based on nanotechnology will be subject to further development in the future. In particular, the unique metabolic vulnerabilities of the triple-negative breast cancer (TNBC) subtype, such as the high expression of GLUT1 and SLC7A11 and the heavy reliance on the pentose phosphate pathway for GSH and NADPH production, make it an optimal candidate for nanomedicines inducing disulfidptosis [192,193]. Exploiting these specific metabolic bottlenecks could facilitate more effective and precise therapeutic outcomes for this highly aggressive subtype.

## 8. Biosafety and Translation

In our view, relying on basic in vitro viability evaluations, such as MTT or CCK-8 assays, creates a false sense of security regarding the biosafety of metal-based nanoparticles. Upon intravenous administration, metallic nanoplatforms rapidly acquire a highly unpredictable biomolecular corona [194,195]. The subsequent “reticuloendothelial system (RES) hijacking” by hepatic Kupffer cells and splenic macrophages indiscriminately clears the vast majority of the injected dose [196,197]. We consistently struggle to predict this anatomical redistribution; it pulls the material far away from the intended breast tumor site, driving inevitable, yet erratically distributed, off-target accumulation.

This unpredictable distribution unmasks specific, element-dependent toxicities that static cell models fail to capture. Despite the translational enthusiasm surrounding iron-induced ferroptosis, the systemic reality is perplexing. Introducing superparamagnetic iron oxide nanoparticles (SPIONs) or ultrasmall superparamagnetic iron oxide nanoparticles (USPIOs) often leads to massive hepatic sequestration [198,199]. The biological dilemma lies in the threshold: at what exact local concentration does physiological iron storage transition into a pathological Fenton-mediated ROS burst? This tipping point appears highly heterogeneous in vivo, frequently resulting in unexpected glutathione (GSH) depletion in non-target hepatocytes. Clinically, this uncontrolled lipid peroxidation translates to sudden, dose-limiting AST and ALT spikes that routinely stall early-phase trials [200,201,202,203], leaving researchers questioning the actual safety margins in diverse patient populations.

Copper interventions face an arguably more immediate, tightly constrained hurdle: a notoriously narrow therapeutic window. As highlighted by current preclinical challenges, it remains unclear how overdose-induced vascular copper toxicity, particularly in cardiac tissues, can be reliably avoided. While intratumoral copper delivery triggers precise cuproptosis, systemic leakage disrupts delicate baseline homeostasis. Even at sub-lethal doses, prematurely released Cu^2+^ ions can rapidly induce hydropic degeneration in periportal hepatocytes or systemic hemolysis [204,205,206]. Defining a universal safety threshold to prevent systemic inflammatory responses without compromising cuproptosis-mediated antitumor efficacy currently seems out of reach.

The regulatory landscape for silver nanoparticles demonstrates the highest level of caution, driven by lingering scientific unknowns. Silver’s primary anti-tumor mechanism, disrupting membrane integrity, is inherently a double-edged sword. We still lack a definitive understanding of why certain sub-20 nm silver nanoparticles inadvertently cross stringent biological boundaries, such as the blood–brain barrier (BBB) [207,208,209], while others do not. This erratic tissue retention and slow clearance precipitate potential systemic toxicity concerns. Consequently, regulatory bodies increasingly demand exhaustive, Good Laboratory Practice (GLP)-compliant multi-organ clearance data (>90 days) before approving Investigational New Drug (IND) applications, as demonstrated by OECD Test Guideline 411 studies [210,211,212,213], effectively pausing many promising platforms.

Bridging the gap between a successful murine xenograft and clinical application requires a fundamental shift in preclinical study design. Future nanomedicine research must move beyond superficial safety claims and confront the uncomfortable uncertainties of longitudinal in vivo pharmacokinetic tracking and long-term elemental clearance.

## 9. From Bench to Bedside

Although nanotechnology has profoundly impacted breast cancer therapy, the current clinical landscape is largely dominated by first-generation nanomedicines, such as PEGylated liposomal doxorubicin (Doxil) and albumin-bound paclitaxel (Abraxane) [214,215]. While these FDA-approved platforms successfully improve pharmacokinetics and reduce systemic toxicity [216], their fundamental mechanism relies on inducing conventional apoptosis. Given that breast cancer cells frequently develop adaptive resistance to apoptotic signaling, the long-term efficacy of these traditional nanodrugs is often compromised. For the broader research community, the seven non-apoptotic regulated cell death pathways (e.g., ferroptosis, cuproptosis, and disulfidptosis) systematically summarized in this review represent far more than isolated preclinical phenomena, they offer critical bypass strategies to overcome the apoptotic resistance inherently limiting current clinical treatments.

Translating these microenvironment-dependent mechanisms into the clinic remains a formidable challenge, yet specific pathways are now yielding valuable patient data. For autophagy, ongoing trials pair inhibitors like chloroquine with albumin-bound paclitaxel to sensitize refractory breast tumors (NCT01446016) [97]. A major practical hurdle here is delivery; achieving true clinical efficacy requires both agents to reliably co-localize within the same hypoxic zones. A distinctly different metabolic strategy exploits iron. Ferumoxytol nanoparticles originally developed for iron deficiency is now being repurposed in metastatic breast cancer cohorts [217,218]. The clinical intent is to use this platform to drive macrophage polarization [217,219] while triggering ferroptosis-driven immune responses within the TME [218,220]. A critical biological caveat remains, however: pushing cells past the ferroptotic threshold assumes the local tumor microenvironment possesses a sufficient oxidative baseline to sustain the Fenton reaction. These early trials provide essential proof-of-concept that non-apoptotic cell death can be therapeutically induced in vivo. At the same time, the biological variability inherent to these single-target interventions highlights a clear clinical need. Overcoming adaptive tumor resistance will likely require next-generation nanoplatforms engineered to engage multiple death cascades simultaneously.

**Figure 5:**
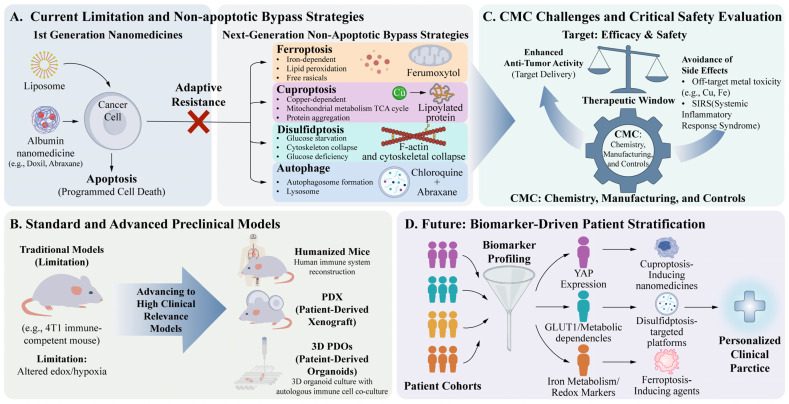
Translational roadmap for next-generation non-apoptotic nanomedicines in breast cancer. (**A**) First-generation nanomedicines primarily rely on conventional apoptosis, which is frequently limited by adaptive tumor resistance. Next-generation platforms exploit non-apoptotic regulated cell death pathways as bypass strategies. (**B**) Advancing these platforms require shifting from conventional murine models to highly clinically relevant systems, including humanized mice, PDXs, and immune-co-cultured 3D PDOs. (**C**) Successful clinical translation is contingent upon fulfilling strict CMC criteria and establishing careful therapeutic windows to avoid off-target metal toxicity and SIRS. (**D**) Future clinical deployment relies on patient stratification driven by specific molecular biomarkers (e.g., YAP for cuproptosis, GLUT1 for disulfidptosis) to achieve personalized nanotherapy. Colored human icons categorize the heterogenous patient cohort into distinct subsets. Black curved arrows trace the clinical decision paths from patient cohorts, through biomarker profiling, leading to customized treatment plans using specific nanomedicine platforms tailored to each subset.

To advance these nanoplatforms toward clinical application, we outline a translational roadmap (Figure 5). A major priority is transitioning from conventional murine models to more clinically relevant systems. While the immunocompetent 4T1 model is widely used for initial immunogenic cell death (ICD) evaluations, its specific macrophage polarization dynamics, pronounced local hypoxia, and altered redox baseline might inadvertently overestimate the efficacy of ROS- or GSH-driven therapies [221,222,223,224,225,226]. Therefore, future validations would benefit from incorporating humanized mice, patient-derived xenografts (PDXs), or 3D patient-derived organoids (PDOs) co-cultured with autologous immune cells, although it remains an open question whether even these advanced systems can fully recapitulate the complex immune microenvironment of human breast tumors. Beyond disease models, clinical translation relies heavily on addressing manufacturing and biosafety challenges. For complex nanocarriers containing transition metals (e.g., copper, iron), meeting strict Chemistry, Manufacturing, and Controls (CMC) criteria is essential but often practically challenging. This includes the ongoing difficulty of establishing careful therapeutic windows to limit the risk of off-target metal toxicity and systemic inflammatory response syndrome (SIRS). Rather than anticipating broad efficacy across all cohorts, the feasibility of these interventions in future trials will likely depend on strict patient stratification guided by specific molecular biomarkers. The deployment of cuproptosis-inducing nanomedicines, for instance, should be guided by specific YAP expression profiles [156], while disulfidptosis-targeted platforms necessitate the profiling of GLUT1 and metabolic dependencies [192]. By adhering to this roadmap, breast cancer nanotherapy can transcend empirical trial-and-error approaches and advance toward personalized clinical practice.

## 10. Conclusions

As a functional position of nanoparticles in breast cancer treatment, it has been evolved beyond the role of mere a drug carrier to complex systems used to orchestrate cell death. This present review elucidates systematically the crosstalk between the apoptosis pathway, autophagy pathway and ferroptosis pathway, and highlights that although nanomaterials can induce synergistic effects by signaling interactions, they can also trigger adaptive resistance in tumor cells. Such dual nature does not only provide a theoretical rationale to the reversal of clinical multidrug resistance but also requires greater precision concerning therapeutic strategies.

Currently, three main bottlenecks impede further development of this field. Firstly, mechanistic studies have been conducted in a fragmented manner and most studies have been confined to the phenotypic validation of individual or paired pathways without any systematic elucidation of the dynamic equilibrium between the death programs; hence, simple inhibition of one node can often trigger therapeutic rebound due to inadvertent pro-survival signals. Secondly, there are significant gaps in biosafety evaluation systems, especially regarding the off-target toxicity and long-term biocompatibility of multi-pathway synergistic activation approaches. Lastly, there remains the challenge of limited translational potential, where existing designs tend to overlook the high levels of molecular heterogeneity of breast cancer. While emerging nanomedicines have begun to target specific receptors or metabolic traits, a systematic integration of distinct molecular subtypes (Luminal, HER2+, and TNBC) into the rational design of regulated cell death remains insufficient. The lack of tailored strategies based on patient stratification continues to hinder clinical translation.

Furthermore, the mechanistic studies and in vivo efficacy evaluations reviewed here rely predominantly on the murine 4T1 breast cancer model (a widely used triple-negative breast cancer model). Although this immunocompetent model is highly valuable for preliminary assessment of immunogenic cell death (ICD) and tumor immune microenvironment remodeling, recognizing its inherent immunological and metabolic limitations relative to human breast cancer is crucial when interpreting the findings discussed herein.

The 4T1 model remains a cornerstone of preclinical breast cancer research, yet its immunological and metabolic fidelity to clinical disease is increasingly debated. Human breast tumors typically present a profoundly immunosuppressive and heterogeneous landscape. In contrast, the robust T-cell responses and distinct macrophage polarization often observed in murine hosts may not adequately capture the translational hurdles of human disease. Metabolic differences further complicate these evaluations. The exceptionally rapid proliferation of 4T1 tumors tends to drive severe local hypoxia and inherently elevated baseline reactive oxygen species (ROS). For nanoparticles designed to trigger ROS bursts, deplete glutathione (GSH), or exploit hypoxia, this hypermetabolic environment can act as a confounding variable. The murine baseline might inadvertently exaggerate the apparent therapeutic response, making it difficult to separate the effects of the nanoparticle design from the model’s pre-existing metabolic bias. Extrapolating such in vivo results to clinical settings generally requires caution. Integrating more clinically reflective systems including humanized mice, patient-derived xenografts (PDXs), and immune-competent 3D organoids (PDOs) can provide a more robust evaluation framework. While no single model is flawless, cross-validating nanoparticle efficacy across these advanced platforms remains a critical step in addressing the complexities of human breast cancer.

Future studies should adopt a mechanism-driven paradigm for rational nanoplatform design. This will require systematic mapping of dynamic cell death regulatory networks through integration of CRISPR screening and single-cell multi-omics, together with the construction of intelligent, microenvironment-responsive systems aided by AI-based nanostructure prediction models, enabling spatiotemporally controlled activation of multiple death pathways. We posit that the future development of organic-inorganic hybrid nano-platforms may transcend the conventional boundaries of material classification. Such intelligent systems can integrate the exceptional biocompatibility and programmable targeting capabilities of organic carriers with the robust catalytic and energy conversion properties of inorganic cores, thereby enabling more precise activation of Regulated Cell Death pathways. It should also be noted that clinical translation of these nanomedicines requires a paradigm shift from solely targeting breast cancer cells to actively remodeling TME. Future nanoplatforms warrant rational design to capitalize on TME hallmarks such as dense stroma, hypoxia, and redox imbalance, thereby dismantling delivery barriers while reactivating immune surveillance and priming tumors for regulated cell death. Pursuing this direction may allow breast cancer nanotherapy to transcend empirical trial-and-error approaches and advance toward a new era of individualized medicine centered on precise decision-making (Table 1).

## Figures and Tables

**Table 1:** Characteristics and mechanistic pathways of nanoparticle-induced regulated cell death (RCD) in breast cancer and cross-tumor models.

Manner of Death	Nanoparticles	Composition & Properties	Model System (New)	Key Targets	Mechanism (Revised & Verified)	Ref.
Pyroptosis	ACS-Z-P/BSM	Metal-based semiconductor, NIR/ultrasound responsive	4T1 (TNBC) model	NLRP3, GSDMD	Elevates intracellular ROS via NIR/ultrasound stimulation, activates the NLRP3 inflammasome, and induces caspase-1-mediated GSDMD cleavage to release IL-1β.	[36,37]
	CS-HAP@ATO	Calcium phosphate/hydroxyapatite, acid-responsive, CD44 targeted	Breast cancer cells	NLRP3, GSDMD	Releases Ca^2+^ and ATO in acidic TME; synergistically causes mitochondrial Ca^2+^ overload and ROS burst, activating NLRP3-caspase-1 axis to cleave GSDMD.	[38]
	TiO2–xFx	Inorganic (Titanium oxide), ultrasound-responsive	4T1 (TNBC) model	Casp-3, GSDME	Generates ROS under ultrasonic cavitation, bypassing the inflammasome to directly activate caspase-3, which specifically cleaves GSDME for pore formation.	[39]
	PLGA (ICG + DAC)	Biomimetic polymeric (PLGA), cell membrane-coated, photothermal	4T1 (TNBC) model	GSDME, Casp-3	DAC up-regulates GSDME; ICG-mediated photothermal effect promotes cytochrome c release, activating caspase-3 to cleave GSDME.	[40]
	NeuNPs (DAC + IR820)	Biomimetic (neutrophil-disguised), photothermal	Breast cancer cells	GSDME, Casp-3	DAC elevates GSDME expression; photothermal effect from IR820 activates caspase-3, causing GSDME cleavage and membrane pore formation.	[41]
	GO (graphene oxide)	Carbon-based	Kupffer cells (KCs)	NLRP3, GSDMD	Activates NADPH oxidase triggering lipid peroxidation, PLC activation, intracellular Ca^2+^ release, and mtROS formation, activating NLRP3 to cleave GSDMD.	[42]
	Biomimetic NPs	Biomimetic cell membrane coating	Breast cancer cells	GSDME, Casp-3	Cell membrane coating facilitates intracellular Ca^2+^ buildup, causing mitochondrial injury, caspase-3 activation, and GSDME-mediated pyroptosis.	[35]
	PIC nanoreactor	Polymeric, ROS-sensitive, enzyme-loaded	Breast cancer cells	GSDME, GPX4, Casp-3	GOx oxidation causes severe oxidative stress and GSH depletion; inhibits GPX4 (ferroptosis) and concurrently stimulates caspase-3 to cleave GSDME (pyroptosis).	[35]
	CaNMs	Calcium-based, pH-responsive	Breast cancer cells	GSDME, Casp-3	Acidic degradation triggers burst release of Ca^2+^, causing mitochondrial overload and caspase-3 activation to cleave GSDME.	[46]
	MCPP NPs	Dual-responsive (ROS/GSH), light-stimulated	Breast cancer cells	GSDME, Casp-3	Degrades in acidic TME; amplifies ROS upon light stimulation, triggering caspase-3 activation and GSDME cleavage.	[50]
	GOx-Mn/HA	Di-enzymatic, hyaluronic acid targeted	Breast cancer cells	NLRP3, GSDMD	GOx consumes glucose to generate H_2_O_2_; Mn nanozymes catalyze ROS production, activating the NLRP3-caspase-1-GSDMD pathway.	[46]
	CSE@PP	Calcium/H_2_S dual-releasing	Tumor cells	NLRP3, GSDMD	Synergistic release of Ca^2+^ and H_2_S induces mitochondrial dysfunction and oxidative stress, activating caspase-1 and promoting GSDMD cleavage.	[48]
	TPP nanomotors	Mitochondria-targeting, NO-generating	General tumor cells	Casp-3, GSDME	Accumulates in mitochondria via TPP; catalyzes NO production, reduces membrane potential, facilitates cyt c release, activating caspase-3 to cleave GSDME.	[49]
	HPPH-ss-NPs	GSH-responsive, photosensitizer-loaded	Breast cancer cells	GSDME, Casp-3	Depletes GSH and induces ROS saturation upon 660 nm irradiation, causing mitochondrial dysfunction, caspase-3 activation, and GSDME cleavage.	[51]
	HM@Ce6@HPB@CS5	Biomimetic, enzyme-responsive, photodynamic	4T1 (TNBC) model	GSDME, GPX4, Casp-3	Generates ROS via Ce6 (PDT) and synergizes with CS-5 to inhibit GPX4, amplifying oxidative stress to activate caspase-3 and cleave GSDME, inducing ICD.	[55]
Apoptosis	PTX-TPP	Polymeric prodrug, mitochondria-targeting	MCF-7 (Luminal A)	Mito, Casp-9/3	Targets mitochondria, disrupts membrane potential, promotes cytochrome c release into cytosol, and activates intrinsic caspase-9 and caspase-3 cascades.	[72]
	ZnO nanofluid	Inorganic (Zinc oxide)	Breast cancer stem-like cells	Mcl-1, Bcl-XL	Inhibits JAK/STAT signaling pathway and downregulates the expression of anti-apoptotic proteins such as Mcl-1 and Bcl-XL.	[75]
	CPNPs	Conjugated polymer, infrared laser-excited	Breast cancer cells	TRPA1, Mcl-1	Modulates TRPA1 channels to inhibit Ca^2+^-calmodulin complex, suppresses Mcl-1, and promotes ROS-mediated apoptosis.	[71]
	TRAIL carrier	Polymeric carrier	Breast cancer cells	DR, Casp-8	Upregulates TRAIL expression, enhances death receptor engagement, and bypasses resistance by activating caspase-8 and caspase-3.	[74]
	mPEG-PCL-DDAB	Polymeric lipid hybrid, siRNA-loaded	MCF-7 (Luminal A)	IGF-1R, αvβ3	Simultaneously silences IGF-1R and integrin αvβ3, blocking survival signaling and leading to cell cycle arrest and apoptosis.	[77,78]
	SLNPs	Solid lipid nanoparticles	Breast cancer cells	JNK, p38 MAPK	Significantly elevates intracellular ROS, triggering JNK and p38 MAPK stress pathways to initiate apoptosis.	[79]
	AgNPs	Inorganic (Silver)	General tumor cells	Cell membrane	Disrupts membrane integrity by generating ROS and lipid peroxidation, causing increased permeability, apoptosis, and necrosis.	[81]
	tBTOma-NPs	Barium titanate, acid-responsive, ultrasound-excited	4T1 (TNBC) model	Physical structure, ROS	Spontaneously assembles in acidic TME; produces elevated ROS under ultrasound and induces apoptosis via direct mechanical damage.	[82]
	IONP-DOX-PolyIC	Iron oxide, endoglin-targeted, pH-responsive	TNBC	TLR3, DNA	Releases DOX to induce DNA damage and ICD; PolyIC activates TLR3 pathway to enhance dendritic cell maturation, synergistically promoting apoptosis.	[83]
	IR783 NPs	Small molecule/dye-based	Breast cancer cells	NQO1, HDAC, EGFR	NQO1 catalyzes β-lapachone to produce ROS; CUDC-101 inhibits HDAC/EGFR, exacerbating DNA damage and ROS-driven drug release to induce ICD and apoptosis.	[85]
Necroptosis	Ag-CS NPs	Inorganic-organic hybrid (Ag-chitosan), shikonin-loaded	TNBC	RIPK3, MLKL	Synergistically upregulates RIPK3 expression and phosphorylates RIPK3/MLKL, promoting MLKL oligomerization and initiating necroptotic ICD.	[66]
	FCA(MET + DOX)	Polymeric nanocarrier, dual-drug loaded	Melanoma (Cross-tumor applicability)	MLKL, GSDMD, Casp-7	Upregulates MLKL expression while simultaneously activating GSDMD and caspase-7, inducing coordinated PANoptosis.	[88]
	BNE-PNP	Polymeric (PLGA), plant extract-loaded	MCF-7 (Luminal A)	TNF-α, p53	Upregulates necroptosis-related genes (TNF-α and p53), inducing programmed necrosis in luminal breast cancer models.	[92]
	PLGA-Dtx	Polymeric (PLGA), docetaxel-loaded	NSCLC	RIPK1, RIPK3	Increases RIPK1 and RIPK3 expression, promoting necroptosis alongside chemotherapeutic stress.	[93]
	mRNA nanocages	Nanocage, mRNA-loaded	Tumor cells	RIPK3	Directly delivers *RIPK3* mRNA to elevate its expression at tumor sites, effectively initiating the necroptotic program.	[94]
	FPS-LNPs	Lipid NPs, nanozyme, shikonin-loaded	Tumor cells	ROS	FePdNZ nanozyme robustly generates ROS, augmenting shikonin-induced necroptosis and activating host immune responses.	[95]
	FSSN	Metal–organic (Fe^3+^-shikonin)	General tumor cells	RIPK1/3, GPX4	Releases Fe^3+^ to elicit necroptosis through Fenton-type processes and ROS generation; synergizes with ferroptosis.	[60]
	MHMO	Mg-doped piezoelectric hydroxyapatite, ultrasound-responsive	TME/Macrophages	DR5, TCR	Ultrasound releases Mg^2+^ (activates TCR) and ROS/Ca^2+^ (upregulates DR5), synergistically driving necroapoptosis and M1 macrophage polarization.	[96]
Autophagy	Tf-Te/HCQ	Transferrin-targeted, HCQ-loaded	Breast cancer cells	Lysosomes, LC3	HCQ alkalinizes lysosomes, blocking autophagic flux; synergistic with Fe^2+^-mediated injury, causing LC3-II and p62 accumulation to sensitize phototherapy.	[103]
	PepLNP	Lipid nanoparticle, PD-L1 targeted	Breast cancer cells	mTOR, PTEN	Mediates PTEN re-expression and PI3K-AKT-mTOR inhibition, eliciting high autophagic stress, CRT exposure, and ICD to reverse immune tolerance.	[107]
	Nano-CUR	Curcumin-loaded nanodelivery system	Breast Cancer Stem Cells	mTOR, AMPK	Activates ROS-AMPK-ULK1 axis and inhibits PI3K-Akt-mTOR; promotes dissociation of Bcl-2 from Beclin-1, enhancing ATG-mediated LC3-II formation.	[110]
	NMK-T-057	Small-molecule complex	4T1 (TNBC) model	Notch, Hes1	Targets the γ-secretase complex, inhibits Notch intracellular domain (NICD) release, and downregulates Hes1 to induce autophagic death.	[112]
	CMB	Inorganic-organic, BSA-coated, catalytic	Tumor cells	ROS	Depletes GSH and catalyzes H_2_O_2_ into massive hydroxyl radicals; ROS bursts trigger pro-death autophagic flux with pronounced autophagosome accumulation.	[113]
	AuNPs	Inorganic (Gold), dual-drug loaded, targeted	Breast Cancer Stem Cells	NCOA4, ferritin	Triggers NCOA4-mediated ferritinophagy to release Fe^2+^, escalating ROS via Fenton reaction; blocks mTORC1 and activates AMPK to induce autophagy.	[116]
Ferroptosis	ssP-tHB@Fe	Polymeric micelles, GSH-responsive, Fe^3+^ loaded	Tumor cells	Iron pool	Disulfide bonds cleaved by high GSH; releases high-valence Fe^3+^ to expand the labile iron pool and promote massive lipid peroxidation.	[137]
	FHA NPs	Hyaluronic acid-iron complex, CD44 targeted	Breast cancer cells	CD44, GPX4	Internalized via CD44; induces ROS and lipid peroxidation through Fenton reaction, downregulates GPX4 expression, triggering ferroptosis.	[131]
	Cu-Pb NPs	Bimetallic	TNBC	GPX4, GSH	Induces synergistic depletion of intracellular GSH, attenuating GPX4 activity, and causing sustained ROS elevation and lipid peroxide accumulation.	[142]
	I@P-ss-FRT	Ferritin-derived, thermal/GSH responsive	Drug-resistant breast cancer	GSH, Iron	Dual response to heat and GSH releases Fe^2+^ and highly depletes GSH, weakening antioxidant defenses to facilitate ferroptosis upon PTT.	[143]
	NBTXR3	Radio-enhancer nanoparticles	Tumor cells	Lysosome	Radiation activation induces lysosomal membrane permeabilization, promoting lipid peroxide accumulation and accelerating ferroptosis.	[145]
	T7-MNT	Magnetic, targeted, magneto-mechanical	Breast cancer cells	Lysosome	Clusters under rotating magnetic field to generate mechanical torque, disrupting lysosomal membranes and triggering massive Fe^2+^ release and lipid peroxidation.	[146]
	TLA	Lysosome-targeted photosensitive agent	Colorectal cancer (Cross-tumor)	Lysosome	Photodynamic therapy induces lysosomal lysis and inhibits autophagy, further potentiating ferroptosis and eliciting ICD.	[148]
Cuproptosis	T-TCu	Copper-based, targeted	Tumor cells	ATP, ATP7A	Attenuates ATP synthesis, indirectly inhibiting ATP7A/ATP7B efflux; accumulated copper catalyzes ROS via Fenton, synergistically downregulating GPX4.	[157]
	CuS/CuO2	Inorganic (Copper sulfide/oxide)	Tumor microenvironment	DLAT, GSH	Releases copper ions, promoting aggregation of lipoylated proteins (DLAT) and GSH depletion, increasing oxidative stress and cell death.	[158,159]
	Cu(I)-BSA	Monoatomic nanozyme, BSA-stabilized	Tumor cells	ATP7A	Catalyzes H_2_O_2_ into ROS, reduces GSH, lowers ATP7A, and maintains Cu(I) intracellularly without oxidation, initiating cuproptosis and enhancing MRI.	[160]
	CuO2-DOX	Hyaluronate-modified, DOX-loaded, GSH-responsive	Breast cancer cells	GSH, ROS	Internalization releases Cu^2+^ and converts H_2_O_2_ into ·OH; DOX elevates ROS while disulfide bonds deplete GSH, synergistically eliciting cuproptosis.	[159]
	Cu-ZnO@PDA	Polydopamine-coated bimetallic, acid-responsive	Breast cancer cells	cGAS-STING	Releases copper/zinc and H_2_O_2_ in acid; causes mitochondrial damage and mtDNA leakage, activating cGAS-STING for DC maturation and T-cell infiltration.	[162]
	PCB	Biomimetic (platelet membrane-coated)	Tumor cells	DLAT, TCA	Releases Cu^2+^ which is reduced by FDX1 to Cu+; binds to lipoylated DLAT, disrupting the TCA cycle while simultaneously suppressing GSH.	[163]
	CussOMEp	Copper-based, omeprazole-loaded, GSH-responsive	Tumor cells (Metastatic)	ATP7A	Generates ·OH, depletes GSH, and utilizes omeprazole to inhibit ATP7A copper efflux, leading to robust intracellular copper accumulation and cuproptosis.	[164]
	D@HCC-CuTH	Hollow calcium carbonate, acid-responsive, disulfiram-loaded	CD44-high breast cancer	Proteasome, DLAT	Generates CuET to inhibit ubiquitin-proteasome (ER stress); free Cu^2+^ induces DLAT aggregation, GSH depletion, and mitochondrial damage.	[171]
Disulfidptosis	GSH-responsive NPs	Polymeric prodrug, GSH-responsive (disulfide)	Breast cancer cells	Disulfide bond	Highly reducing TME cleaves disulfide bonds to release paclitaxel and 30-HPT, causing drug-specific cytotoxicity and inhibiting tumor metastasis.	[179]
	PSSMAL	Prodrug, albumin-binding, GSH-responsive	Tumor cells	Albumin	Accumulates via albumin binding; reducing TME cleaves disulfide bonds to release active paclitaxel and induce cell death.	[180]
	IrssQu	Dual-drug conjugate, GSH-responsive	Drug-resistant tumors	P-gp	Disulfide bonds cleaved by GSH release irinotecan and quinine; inhibits P-glycoprotein efflux to reverse multidrug resistance.	[181]
	d-SN38@NPs	Prodrug assembly, iRGD-targeted, photosensitizer-loaded	4T1 (TNBC) model	GSH, ROS	Elevated GSH cleaves disulfide bonds releasing SN38 (topo I inhibition); Ce6 PDT generates ROS, exacerbating disulfide stress and apoptosis.	[182]
	PEI-SS-VES	Polymeric, targeted, light-activated plasmid	4T1 (TNBC) model	EF2	GSH-cleaved release; blue light initiates DTA expression, inhibiting eukaryotic elongation factor 2 (EF2) to block protein synthesis and induce apoptosis.	[185]
	CCD@RF	Multi-drug loaded, metal-coordinated	Tumor cells	GLUT1, F-actin	Downregulates GLUT1/NADPH and exhausts GSH (via NF-κB inhibition/Cu^2+^ reduction), precipitating disulfide stress and F-actin cytoskeletal collapse.	[176]
	FeOOHFe-ApAuNSs	Inorganic (Au/Fe), GOx-mimetic	Ovarian cancer (Applicable to TNBC)	SLC7A11, Actin	GOx-mimetic activity inhibits NADPH and cystine conversion; exorbitant cystine triggers actin disulfide cross-linking, initiating disulfidptosis and ferroptosis.	[187]
	CYBC NPs	Biomimetic membrane-coated, BAY-876 loaded	TNBC	GLUT1, NADPH	Uses BAY-876 to inhibit glucose uptake while supplying exogenous cystine; induces intense disulfide stress and F-actin collapse via metabolic heterogeneity.	[192]

## Data Availability

No new data were created or analyzed in this study.

## Abbreviations

30-HPT: 30-hydroxypterostilbene; AKT: Protein kinase B; AMPK: AMP-activated protein kinase; ATG: Autophagy-related gene/protein; ATO: Atorvastatin; ATP: Adenosine triphosphate; ATP7A: ATPase copper transporting alpha; AuNPs: Gold nanoparticles; BAY-876: A selective GLUT1 inhibitor; Bcl-2: B-cell lymphoma 2; Bcl-XL: B-cell lymphoma-extra large; BSA: Bovine serum albumin; Casp-1/3/7/8/9: Caspase-1/3/7/8/9; CD44: Cluster of differentiation 44; Ce6: Chlorin e6; cGAS: Cyclic GMP-AMP synthase; CRT: Calreticulin; CuET: Copper diethyldithiocarbamate; CUR: Curcumin; cyt c: Cytochrome c; DAC: Decitabine; DC: Dendritic cell; DLAT: Dihydrolipoamide S-acetyltransferase; DOX: Doxorubicin; DR/DR5: Death receptor/Death receptor 5; DTA: Diphtheria toxin A chain; Dtx: Docetaxel; EF2: Eukaryotic elongation factor 2; EGFR: Epidermal growth factor receptor; ER: Endoplasmic reticulum; F-actin: Filamentous actin; FDX1: Ferredoxin 1; FePdNZ: Iron-palladium nanozyme; GLUT1: Glucose transporter 1; GO: Graphene oxide; GOx: Glucose oxidase; GPX4: Glutathione peroxidase 4; GSDMD: Gasdermin D; GSDME: Gasdermin E; GSH: Glutathione; HA: Hyaluronic acid; HCQ: Hydroxychloroquine; HDAC: Histone deacetylase; Hes1: Hes family bHLH transcription factor 1; ICD: Immunogenic cell death; ICG: Indocyanine green; IGF-1R: Insulin-like growth factor 1 receptor; IL-1β: Interleukin-1 beta; IONP: Iron oxide nanoparticles; iRGD: Internalizing RGD peptide; JAK/STAT: Janus kinase/signal transducers and activators of transcription; JNK: c-Jun N-terminal kinase; KCs: Kupffer cells; LC3: Microtubule-associated protein 1A/1B-light chain 3; LMP: Lysosomal membrane permeabilization; LNPs: Lipid nanoparticles; MAPK: Mitogen-activated protein kinase; MCF-7: Michigan Cancer Foundation-7 (Breast cancer cell line); Mcl-1: Myeloid cell leukemia 1; MET: Metformin; Mito: Mitochondria; MLKL: Mixed lineage kinase domain-like protein; MRI: Magnetic resonance imaging; mRNA: Messenger RNA; mtDNA: Mitochondrial DNA; mTOR: Mammalian target of rapamycin; mTORC1: Mammalian target of rapamycin complex 1; mtROS: Mitochondrial reactive oxygen species; NADPH: Nicotinamide adenine dinucleotide phosphate; NCOA4: Nuclear receptor coactivator 4; NF-κB: Nuclear factor kappa B; NICD: Notch intracellular domain; NIR: Near-infrared; NLRP3: NLR family pyrin domain containing 3; NO: Nitric oxide; NPs: Nanoparticles; NQO1: NAD(P)H quinone dehydrogenase 1; NSCLC: Non-small cell lung cancer; p53: Tumor protein p53; PANoptosis: Pyroptosis, Apoptosis, and Necroptosis; PD-L1: Programmed death-ligand 1; PDT: Photodynamic therapy; P-gp: P-glycoprotein; PI3K: Phosphoinositide 3-kinase; PLC: Phospholipase C; PLGA: Poly (lactic-co-glycolic acid); PolyIC: Polyinosinic:polycytidylic acid; PTEN: Phosphatase and tensin homolog; PTT: Photothermal therapy; PTX: Paclitaxel; RIPK1: Receptor-interacting protein kinase 1; RIPK3: Receptor-interacting protein kinase 3; ROS: Reactive oxygen species; siRNA: Small interfering RNA; SLC7A11: Solute carrier family 7 member 11; SLNPs: Solid lipid nanoparticles; SN38: 7-ethyl-10-hydroxycamptothecin; STING: Stimulator of interferon genes; TCA: Tricarboxylic acid; TCR: T-cell receptor; Tf: Transferrin; TLR3: Toll-like receptor 3; TME: Tumor microenvironment; TNBC: Triple-negative breast cancer; TNF-α: Tumor necrosis factor-alpha; topo I: Topoisomerase I; TPP: Triphenylphosphonium; TRAIL: Tumor necrosis factor-related apoptosis-inducing ligand; TRPA1: Transient receptor potential ankyrin 1; ULK1: Unc-51 like autophagy activating kinase 1.
